# Subarachnoid hemorrhage: epidemiology, risk factors, pathogenesis, and clinical therapies

**DOI:** 10.1186/s43556-026-00455-y

**Published:** 2026-04-27

**Authors:** Hangyu Shen, Xu Yan, Mingyue Zhao, Jie Sun, Yi Huang

**Affiliations:** 1 Ningbo Key Laboratory of Nervous System and Brain Function, Department of Neurosurgery, The First Affiliated Hospital of Ningbo University, Ningbo, Zhejiang 315010 China; 2 Key Laboratory of Precision Medicine for Atherosclerotic Diseases of Zhejiang Province, Ningbo, Zhejiang 315010 China

**Keywords:** Subarachnoid hemorrhage, Epidemiology, Risk factors, Early brain injury, Delayed cerebral ischemia, Therapeutic targets

## Abstract

Subarachnoid hemorrhage (SAH) is an extremely lethal cerebrovascular emergency that can lead to severe long term neurological deficits. Modern research evidence indicates that the incidence of SAH varies significantly across different regions and populations, and this variation is determined by the interaction between non—modifiable factors (such as age, gender, family history, and genetic susceptibility) and modifiable factors (such as hypertension, smoking, drug use, and metabolic disorders). Advancements in vascular biology and genomics further suggest that extracellular matrix instability, endothelial dysfunction, chronic inflammation, and population—specific genetic variations play crucial roles in aneurysm formation and rupture. After aneurysm rupture, SAH triggers a biphasic injury cascade. Early brain injury (EBI) occurs within minutes to hours, manifesting as a sudden increase in intracranial pressure, systemic hypoperfusion, blood brain barrier disruption, neuroinflammation, cerebral edema, and metabolic failure. These early disturbances create favorable conditions for delayed cerebral ischemia (DCI), which occurs several days later due to microvascular dysfunction, impaired neurovascular coupling, cortical spreading depolarization, and progressive immunometabolic changes. Understanding this mechanistic continuum is of great significance for early detection and selection of treatment targets. Recent advancements in neuroimaging, biomarker development, and multimodal monitoring have improved diagnostic accuracy, while current management measures include rapid aneurysm fixation, optimized intensive care, and emerging neuroprotective strategies. Despite these advancements, treatment outcomes remain suboptimal, highlighting the need for further improvements in risk stratification, mechanism-based interventions, and population specific preventive measures. This review synthesizes the latest knowledge on the epidemiology, risk factors, pathogenesis, and clinical management of SAH to support a more comprehensive research and care framework.

## Introduction

Subarachnoid hemorrhage (SAH) is a life threatening cerebrovascular disease characterized by sudden onset, rapid deterioration of neurological function, and long term severe sequelae. Although it accounts for only 2% to 5% of all stroke cases, SAH contributes significantly to the increase in global disability adjusted life years because this disease often occurs in younger individuals and leads to persistent cognitive impairment, functional dependence, and decreased quality of life [1–3]. Contemporary epidemiological studies have revealed significant differences in the incidence of SAH among different regions, different populations, and different races, and people are increasingly recognizing its huge social and economic burden [4–7]. These observations indicate that SAH is a major global health challenge and emphasize the need to improve early identification, prevention, and management strategies in the acute phase.

The clinical trajectory of SAH is shaped by both the primary hemorrhagic insult and a cascade of secondary injuries that unfold within minutes and extend over days to weeks. Among these complications, cerebral vasospasm (CVS) and delayed cerebral ischemia (DCI) remain the most feared, accounting for a large proportion of neurological deterioration and poor functional outcomes despite timely aneurysm repair [8–12]. Advances in aneurysm securing techniques particularly endovascular coiling and microsurgical clipping along with refinements in neurocritical care have reduced early mortality; however, improvements in long-term functional outcomes have been modest [13, 14]. Diagnostic practice continues to rely heavily on computed tomography (CT) and clinical grading scales, which, while essential for acute assessment, provide limited insight into the evolving pathophysiological processes that drive secondary injury and exhibit variability in prognostic accuracy [15–18].

An increasing amount of mechanistic evidence indicates that SAH triggers a staged but persistent pathological process, which includes early brain injury (EBI) and DCI. EBI occurs within minutes and includes sudden increase in intracranial pressure, global cerebral hypoperfusion, disruption of the BBB, neuroinflammation, brain edema, mitochondrial dysfunction, and various forms of programmed cell death [19–21]. These early disorders prepare the brain for DCI, which is not only caused by large artery vasospasm but also mediated by microvascular dysfunction, impaired neurovascular coupling, cortical diffuse depolarization, and immune metabolic remodeling. Understanding the mechanism of this continuous process is crucial for guiding therapeutic interventions beyond aneurysm repair, which should target downstream injury pathways [22–24].

In this context, proteomics and other high throughput molecular platforms have become complementary tools for describing the dynamic biological responses following SAH. Although numerous candidate biomarkers have been identified, including inflammatory mediators, extracellular matrix regulators, and neuroinjury markers, they have potential correlations in risk stratification or brain injury prediction [25–29], yet their application in practice is still limited by methodological heterogeneity and limited multicenter validation [30–32]. Therefore, these molecular tools are best regarded as supplements to the established clinical and imaging frameworks rather than definitive diagnostic or prognostic tools.

In this context, this review synthesizes contemporary evidence on the epidemiology, risk factors, pathophysiological mechanisms, clinical diagnosis, and treatment strategies of SAH. Special emphasis is placed on the mechanistic basis of EBI and DCI as well as the latest advancements in clinical management. The review also discusses the emerging but still developing roles of proteomics and multi omics methods, highlighting their potential contributions in precision medicine and the challenges that must be overcome to achieve effective clinical translation.

## Epidemiology and global burden of SAH

### Incidence, prevalence, and temporal trends

SAH accounts for only 2%—5% of all strokes, yet its clinical and societal impact is disproportionately severe due to high case fatality and long term disability. According to the Global Burden of Disease (GBD) 2021 report, SAH resulted in approximately 700,000 new cases, nearly 8 million prevalent cases, about 350,000 deaths, and more than 10 million disability adjusted life years (DALYs) worldwide in 2021 [33]. The estimated global age standardized incidence rate (ASIR) was 8.3 per 100,000 person years [34].

The trend of time shows a diverse global situation. The incidence rate in high income countries has gradually decreased, which is partly attributed to the improvement in hypertension management, the reduction in smoking rates, and the progress in screening and treatment of unruptured aneurysms. However, the rate of decline in incidence is slower than that of mortality or the rate of disability adjusted life years decline, indicating that the disease burden still persists. On the contrary, in many low income and middle income countries (LMICs), the incidence rate seems to remain stable or even increase, mainly driven by population aging, urbanization, and improved diagnosis and identification [35].

Substantial national variation persists. For example, recent population-based research from Korea reported regional spontaneous SAH incidences ranging from 9.0 to 15.7 per 100,000 person years higher than global averages [36]. These differences underscore that SAH remains a critical public health challenge, with global epidemiological dynamics influenced by demographic shifts, healthcare access, and emerging risk factor distributions.

### Demographic, regional, and molecular epidemiological variations

**Fig. 1:**
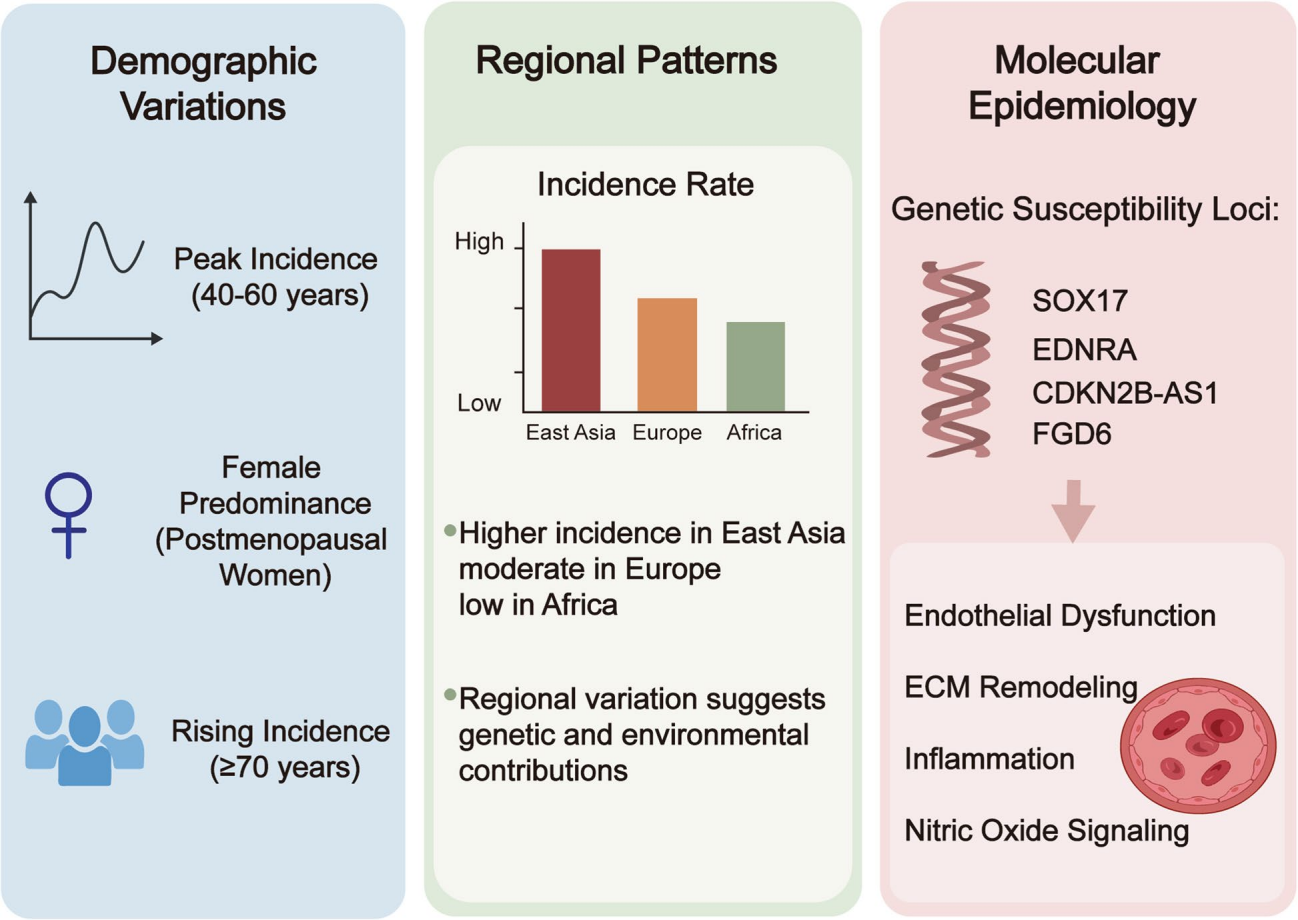
Demographic, regional, and molecular epidemiological characteristics of SAH. The incidence of diseases is influenced by age, gender, and factors related to aging. The incidence varies across different regions and is driven by genetic susceptibility as well as downstream mechanisms including endothelial dysfunction, inflammation, extracellular matrix remodeling, and nitric oxide signaling

Demographic and regional factors exert substantial influence on the risk and burden of SAH (Fig. 1). The disease typically presents approximately a decade earlier than ischemic stroke, with the peak incidence occurring between 40 and 60 years of age. Women are consistently more affected than men, particularly after menopause, suggesting that estrogen deficiency may contribute to vascular fragility and aneurysm susceptibility. Recent epidemiological evidence indicates that although age standardized SAH incidence rates have declined in many countries, older age groups (≥ 70 years) are showing a sharper absolute increase in incident cases, likely reflecting growing life expectancy and shifting population structures [37]. A recent global analysis confirmed that while standardized rates have fallen worldwide, the overall burden remains disproportionately high in lower SDI regions and in specific age groups [35]. Sex based prognosis also differs: female SAH patients exhibit higher rates of DCI, hydrocephalus, and unfavorable 3 month outcomes compared with males [38]

The regional and social demographic index (SDI) gradient further influences the global distribution of SAH. The total number of cases and the burden of DALYs in low SDI regions are significantly higher, even though the age standardized incidence rate seems stable or only shows a slight decrease [35]. East Asia, high income Asia Pacific regions, and some countries in Oceania reported the highest age standardized incidence rates (approximately 14 cases per 100,000 people) in 2021. In contrast, the mortality and DALYs figures in sub-Saharan Africa and Central Asia show a slow decline or, in some cases, a continuous increase, highlighting the influence of medical conditions, lifestyle factors, and vascular risk characteristics [39, 40]. Gender related patterns also vary across different regions: globally, the age-standardized mortality and DALYs indicators for most age groups are slightly higher in men than in women, but in specific elderly populations and certain geographical areas, the incidence or mortality rates of women are close to or exceed those of men, highlighting the important interaction between age and gender in the epidemiology of SAH [34, 41, 42].

Beyond traditional demographic determinants, molecular epidemiology has begun to illuminate genetic and ancestry specific risk differences that may underlie observed epidemiological patterns. Several genome wide association studies have identified susceptibility loci associated with intracranial aneurysm formation and rupture such as variants near SOX17, EDNRA, CDKN2B-AS1, and FGD6 which exhibit different allele frequencies across ethnic populations [43, 44]. These variations may help explain the higher SAH incidence observed in East Asian and Finnish populations compared with Western European or African cohorts [45]. For example, risk alleles related to endothelial dysfunction, extracellular matrix remodeling, or vascular smooth muscle homeostasis appear more prevalent in certain ancestries, suggesting molecular contributions to population level disparities. Moreover, interactions between genetic predisposition and modifiable exposures (e.g., smoking, hypertension, alcohol intake) may differ by ethnicity, shaping both aneurysm biology and rupture risk [43].

Recent studies at the population level have further demonstrated that variations related to inflammatory pathways, nitric oxide signaling, and lipid metabolism not only may affect the susceptibility to SAH, but also may influence the conditions of complications such as DCI or cerebral infarction [46]. These findings indicate that the regional and population differences in the risk of SAH are not solely driven by environmental or socioeconomic factors, but may also reflect the specific molecular structures of certain races, which make individuals more prone to vascular instability and lead to different clinical trajectories. With the continuous expansion of global multi omics datasets, combining molecular epidemiology with traditional demographic assessment is expected to enhance the ability of risk stratification, optimize prevention strategies for specific populations, and reveal the underlying mechanisms of the burden of SAH worldwide [47].

### Global disability and health economic burden

Despite substantial improvements in contemporary neurocritical care, SAH continues to impose a profound global disability burden. Mortality remains between 30%—40%, and among survivors, approximately half experience persistent neurological deficits, cognitive impairment, or reduction in functional independence [48, 49]. Many survivors face prolonged rehabilitation needs, decreased participation in daily life, and difficulties returning to work or prior social roles.

The global disability adjusted life years caused by SAH have remained at a level of several million, reflecting both a high premature mortality rate and a long term disability status [34]. This burden is particularly severe in low and middle income countries, as factors such as delayed patient visits, difficulty in accessing specialized neurovascular treatment, and resource scarcity exacerbate the adverse consequences in these countries.

The economic burden varies significantly among different healthcare systems, mainly due to the high direct medical costs (hospitalization, neuro intensive care, endovascular or surgical aneurysm repair, rehabilitation treatment) and the indirect social costs resulting from decreased productivity, long term care needs, and reduced labor participation. Health economic analyses from multiple regions consistently show that SAH is one of the costliest medical conditions for patients in the context of neurological diseases [50, 51]. Overall, these research results indicate that SAH remains a major global health issue. The demographic changes, persistent diseases, and significant economic impacts collectively highlight the urgent need to improve risk stratification, conduct early biomarker based complication detection, and adopt evidence based strategies to alleviate the global burden of disease.

## Risk factors for SAH: from clinical to molecular determinants

**Fig. 2:**
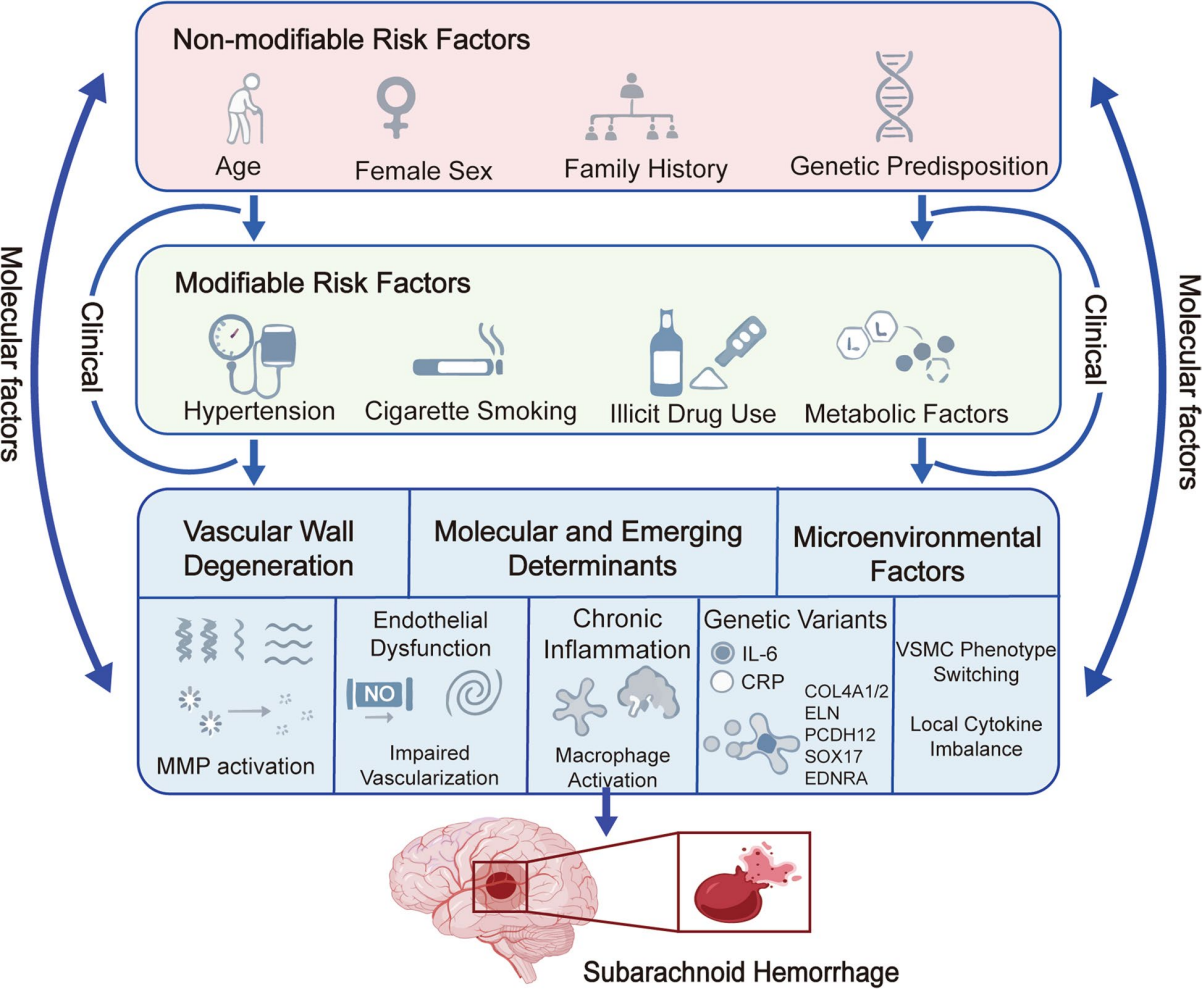
Risk factors and mechanisms in SAH**.** The irreversible risk factors and the modifiable risk factors for subarachnoid hemorrhage jointly act on the degeneration of the vascular wall, driven by endothelial dysfunction and adaptive remodeling. These processes are further exacerbated by molecular and microenvironmental changes, including inflammation, macrophage activation, genetic susceptibility, vascular smooth muscle cell phenotypic transformation, and cytokine imbalance, ultimately promoting the formation, progression, and rupture of aneurysms

The formation of SAH is the result of complex interactions among various factors such as clinical, environmental and molecular ones. Traditional epidemiological studies have identified a series of factors related to demographics and lifestyle that determine the baseline susceptibility; however, the latest advancements in genetics, vascular biology and multi omics analysis increasingly indicate that the risk of SAH is also controlled by the intrinsic vulnerability of the cerebral vessels, including the instability of the extracellular matrix (ECM), endothelial dysfunction and inflammation driven degeneration of the vascular wall. Integrating these multi-level determinants from biological characteristics to molecular pathology can provide a more comprehensive perspective on the formation and rupture of intracranial aneurysms (Fig. 2).

### Non-modifiable factors: genetic background, age, and sex

Non-modifiable risk factors play a crucial role in determining the susceptibility to SAH. Aging is associated with the cumulative remodeling of blood vessels, including the disruption of elastic fiber structure, the interruption of collagen cross linking, and the transformation of vascular smooth muscle cells (VSMCs) phenotype, all of which increase the vulnerability of the aneurysm wall [52, 53]. Female gender has consistently been shown to bring a higher risk, with the incidence of SAH in women being 1.3—1.6 times that of men [52]. Estrogen deficiency, especially after menopause, reduces the activity of endothelial nitric oxide synthase (eNOS), enhances inflammatory signaling, and disrupts the homeostasis of the ECM, all of which increase the possibility of aneurysm formation and rupture. Family history is one of the strongest predictors of SAH. People with first degree relatives who have intracranial aneurysms or aneurysmal SAH have a risk that is 3—5 times higher, with an estimated heritability of 10%—20% [54]. Genetic association studies have identified some variants that regulate the integrity of extracellular matrix structure, inflammatory response, and vascular remodeling, all of which are related to this genetic susceptibility. For example, polymorphisms in genes encoding components of the basement membrane, such as COL4A1/2, weaken the resistance of capillaries and arterial walls [59]. Variants of IL6 and MMP9, which are key regulators of inflammation and proteolytic activities, are associated with an increased risk of aneurysm formation and rupture [55].

The new genetic research evidence also indicates that there are other factors besides those mentioned above that increase the risk of SAH [56]. The original cadherin 12 gene (PCDH12) is related to the stability of endothelial cell adhesion and may affect the vascular response to pulsatile pressure. Mutations in the ELN gene, which encodes elastin, can affect the elasticity of the vascular wall, and rare mutations can reduce the ability of blood vessels to adapt to fluctuations in hemodynamic load [57, 58]. Population wide genome studies across different populations have shown that single nucleotide polymorphisms related to inflammation and extracellular matrix regulation have different effect sizes in Asian and European populations, which may explain the differences in the incidence of SAH in different regions [43, 59]. In summary, these genetic factors contribute to the "baseline vulnerability phenotype", that is, the vascular wall is more susceptible to the effects of hemodynamics and inflammation.

### Modifiable factors: smoking, hypertension, and mechanistic insights

Among modifiable factors, hypertension and smoking are the most significant and clinically actionable determinants of SAH risk [60, 61].

Hypertension chronically increases mechanical stress on intracranial arteries. Sustained elevations in blood pressure have been associated with arterial remodeling in intracranial vessels, including structural thickening and wall remodeling [62, 63]. These changes likely involve maladaptive remodeling processes such as VSMC migration and ECM/elastin collagen architecture disruption, as shown in hypertensive vascular remodeling models [64].Moreover, hemodynamic strain may activate mechanosensitive molecules such as Piezo1, which has been demonstrated to mediate Ca^2^⁺ influx in vascular smooth muscle cells and trigger arterial remodeling in response to biomechanical stress [65, 66].

Smoking causes vascular toxicity through multiple mechanisms. The oxidative substances produced by tobacco can lead to endothelial dysfunction, reduce the bioavailability of nitric oxide (NO), and promote uncoupling of eNOS [67]. Smoking also enhances the infiltration of macrophages, promoting the transformation of cells to the destructive M1 phenotype, activating the NLRP3 inflammasome, and increasing the secretion of matrix metalloproteinases (MMP) [68]. These processes accelerate the degradation of the extracellular matrix, leading to apoptotic loss of vascular smooth muscle cells, which are the key structural components maintaining the tension of the aneurysm wall. In addition to its effects on the vascular system, smoking is also associated with an accelerated growth rate, increased occurrence, and increased risk of rupture of aneurysms, and is not related to blood pressure [69].

Excessive alcohol consumption and the use of recreational drugs such as cocaine and amphetamines will further increase the risk of SAH. The main mechanism is the cause of acute hypertension surge and direct toxicity to the cerebral vessels [70–72]. Although metabolic disorders such as hyperlipidemia, diabetes, and obesity have inconsistent independent associations with SAH, when combined with smoking or hypertension, they may produce a synergistic effect, thereby exacerbating oxidative stress and mild vascular inflammation [73, 74].

### Molecular and emerging risk factors: vascular wall degeneration, genetic variants, and microenvironmental dysregulation

The rapid advancements in single cell transcriptomics, spatial analysis, and large scale population genetics studies have revealed that the molecular determinants leading to the formation and rupture of aneurysms present an increasingly broad range [75, 76]. These factors include vascular wall degeneration, genetic susceptibility, and local microenvironmental imbalance, providing crucial mechanistic insights into how epidemiological risks translate into the vulnerability of cerebral vascular structures [77, 78].

The degeneration of the vascular wall is a core pathological process. The excessive activation of matrix metalloproteinases (especially MMP-2 and MMP-9) accelerates the degradation of elastin and collagen, gradually weakening the tensile strength of the arterial wall [77]. At the same time, the continuously activated JAK/STAT and NF-κB signaling pathways promote chronic macrophage infiltration and the continuous release of pro-inflammatory cytokines, thereby exacerbating local inflammatory damage [79]. The disruption of the TGF-β/SMAD regulatory pathway also leads to adverse changes in adaptive ECM remodeling, and vascular smooth muscle cells will shift from a contracting state to a synthesizing state, increasing protease secretion and reducing structural support [80, 81]. These interwoven changes transform the originally stable artery into a mechanically fragile vessel, becoming increasingly prone to rupture under physiological hemodynamic pressure.

Genetic susceptibility may operate in parallel with the degenerative pathway. In human studies, variations in genes related to extracellular matrix (such as COL4A1/COL4A2 and MMP9) are most strongly associated with the risk of intracranial aneurysms, suggesting that certain individuals have structural/matrix fragility [55]. At the same time, it is not yet clear whether other genes (such as those encoding elastin, matrix cross linking enzymes, or cell adhesion molecules) may regulate the elasticity and mechanical transduction of the vascular wall, thereby leading to a "baseline fragility phenotype". This conceptual model awaits validation through the integration of genetic, transcriptomic, and biomechanical data in large-scale, multi-omics studies [43, 77].

The vascular microenvironment further integrates systemic and local influencing factors, which can regulate the vulnerability of aneurysms. Individuals in a high risk state typically exhibit a low level pro-inflammatory state, characterized by elevated levels of C-reactive protein, interleukin-6 and related biomarkers in the circulation. This is not only related to the clinical outcomes after SAH but may also reflect the inflammatory environment before rupture [82, 83]. Symptoms caused by endothelial dysfunction, such as impaired nitric oxide signaling, excessive reactive oxygen species, and impaired barrier integrity, reduce the vascular ability to resist hemodynamic pressure [84, 85]. Hormonal factors, especially estrogen deficiency, exacerbate oxidative stress and disrupt the renewal of extracellular matrix, thereby leading to the progression of aneurysms and the risk of rupture [86]. Genetic connective tissue diseases such as Ehlers Danlos syndrome and polycystic kidney disease further illustrate the extreme situation of extracellular matrix fragility, highlighting the interaction between genetic structure and vascular biomechanical failure [87]. These molecular and microenvironmental factors collectively form a comprehensive framework that links intrinsic vascular biology with the clinical epidemiology of SAH [88].

## Pathophysiological mechanisms of SAH

SAH triggers a series of complex pathological and physiological changes that continue to evolve over time and affect both early and late brain injuries [89]. This section will outline the key mechanisms underlying subarachnoid hemorrhage, focusing on the immediate effects of mechanical and biochemical damage caused by the hemorrhage, as well as the dynamic interactions among major pathological processes such as inflammation, oxidative stress, disruption of the blood–brain barrier, and vascular dysfunction [90, 91]. Understanding these interrelated mechanisms is crucial for elucidating disease progression and identifying potential therapeutic targets.

### Immediate brain injury: mechanical and biochemical insults

The initial phase following aneurysmal rupture is dominated by acute mechanical and biochemical insults that together establish the substrate for subsequent secondary injury. The sudden extrusion of arterial blood into the subarachnoid space produces an abrupt rise in intracranial pressure and a commensurate fall in cerebral perfusion pressure often resulting in transient global cerebral ischemia and pronounced metabolic disturbance within minutes after the hemorrhagic event [90, 92]. This hemodynamic derangement can lead to neuronal depolarization, ionic imbalance, energy failure, and sets in motion cell‑intrinsic injury cascades. Concurrently, exposure to blood‑derived mediators (e.g., hemoglobin breakdown products, iron, free radicals) initiates oxidative stress, BBB disruption, neuroinflammation, and cytotoxicity processes believed to amplify the initial insult and contribute to EBI and downstream complications [93, 94].

Among these mediators, hemoglobin and its degradation products are central drivers of early biochemical toxicity. Heme and iron catalyze Fenton chemistry and other redox reactions that precipitate excessive production of reactive oxygen species (ROS), lipid peroxidation and oxidative damage to membranes, proteins and nucleic acids [90]; this oxidative milieu contributes directly to mitochondrial dysfunction and early neuronal injury [95, 96]. Concurrently, blood constituents in the subarachnoid compartment activate innate immune sensors: damage associated molecular patterns (DAMPs) derived from erythrocyte breakdown and injured parenchymal cells engage microglia and infiltrating leukocytes, provoking an immediate inflammatory response that further compromises neuronal and vascular integrity [90, 97–99].

Proteomic investigations of CSF and peri hematomal tissue in early and subacute phases following aneurysmal SAH have identified distinct molecular signatures indicative of inflammatory activation, oxidative injury, and ECM remodeling. In a recent mass‑spectrometry study of 152 SAH patients, differentially expressed proteins associated with poor outcome included those involved in inflammatory pathways and oxidative stress [100]. Consistent with this, other studies have documented upregulation of neutrophil‑related alarmins (e.g. S100A9), matrix metalloproteinases (e.g. MMP‑9), and proteins related to astrocytic or endothelial injury in CSF, suggesting early activation of proteolytic and inflammatory cascades [101]. These findings support a model in which blood‑derived mediators trigger proteolytic degradation of tight junction and basal lamina components, compromise blood brain barrier (BBB) integrity, and promote leukocyte infiltration and perivascular inflammation processes that may underlie early BBB dysfunction, interstitial fluid accumulation, and secondary brain injury after SAH [102].

The confluence of ischemia, oxidative stress and early inflammation manifests clinically as cerebral edema, impaired autoregulation, and early neurological deterioration [90]. Mitochondrial impairment and calcium dysregulation trigger apoptotic and necrotic cascades in vulnerable neuronal populations, whereas iron mediated lipid peroxidation has been implicated in ferroptotic processes that may uniquely contribute to SAH related parenchymal injury [103, 104]. Importantly, the molecular events of this immediate phase are temporally dynamic: many protein mediators rise within hours and evolve over the first 72 h, highlighting a critical therapeutic window in which interventions aimed at limiting oxidative injury, stabilizing the BBB, modulating protease activity or blunting early inflammatory activation may have the greatest potential to mitigate downstream secondary insults.

In summary, the immediate brain injury following SAH reflects an integrated response to abrupt hemodynamic compromise and a milieu of blood derived biochemical toxins. Proteomic profiles obtained in the hyperacute and early phases corroborate the central roles of oxidative stress, protease mediated matrix degradation, glial and endothelial activation, and innate immune engagement processes that collectively convert a mechanically induced ischemic insult into a self-propagating molecular cascade that shapes early outcome and primes the brain for delayed complications [105, 106].

### Mechanistic interplay in SAH

**Fig. 3:**
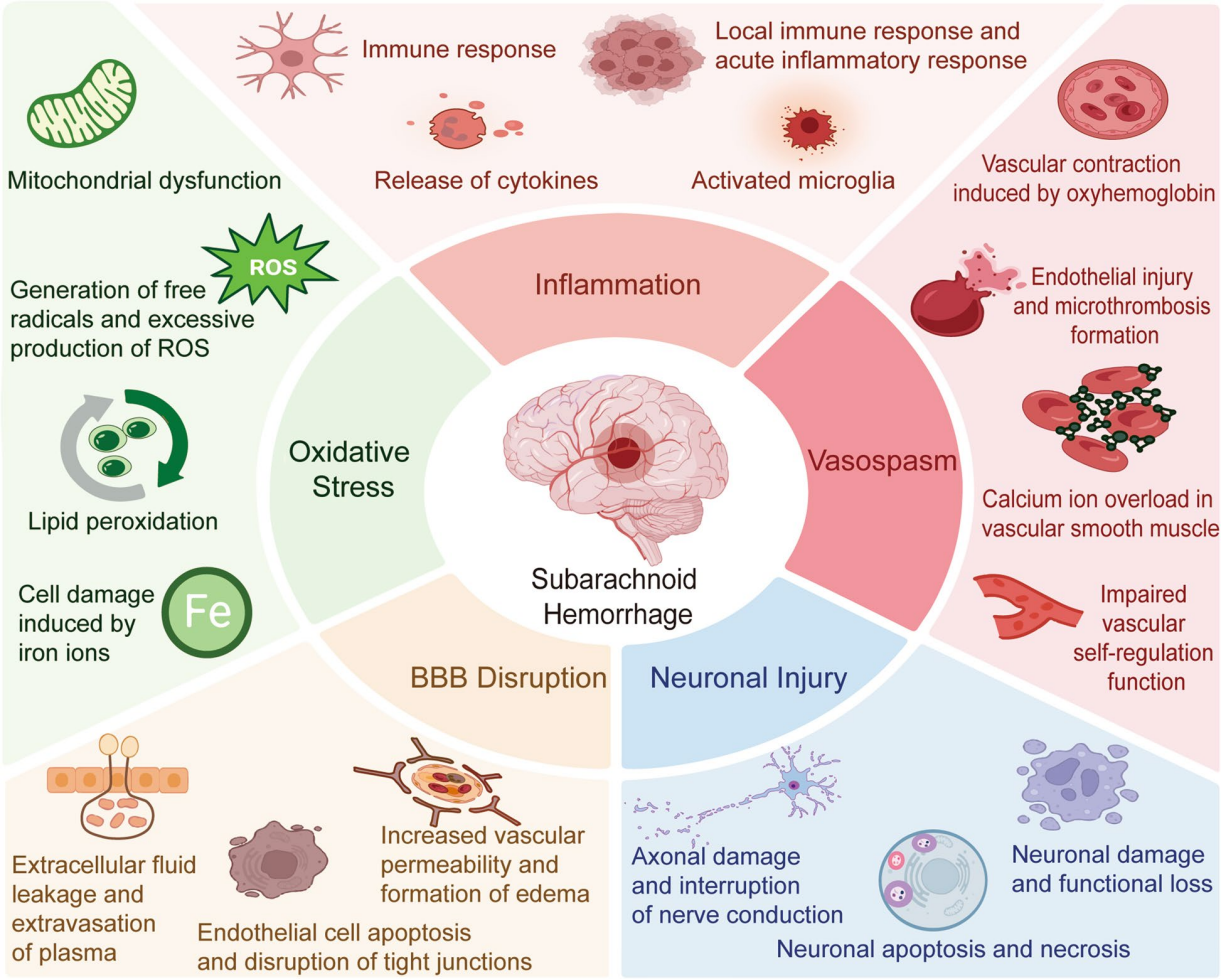
Pathophysiological mechanisms in SAH**.** SAH involves complex and interrelated pathophysiological mechanisms. Key processes include oxidative stress, neuroinflammation, cerebral vasospasm, disruption of the BBB, and neuronal damage, all of which are regarded as the main factors contributing to secondary brain injury. These mechanisms interact and cooperate with each other, jointly driving the progression of brain injury after subarachnoid hemorrhage

Brain injury following SAH is a multifactorial and complex pathological process, which is generally divided into two stages: EBI and DCI [107, 108]. After blood and its breakdown products enter the subarachnoid space, a cascade of cellular and molecular responses is rapidly triggered. The interaction and change process between these stages can be illustrated by the following Fig. 3. Among these, inflammation, oxidative stress, CVS, BBB disruption, and neuronal apoptosis interact and amplify each other, forming a sustained network of neurovascular injury that serves as the key pathological basis determining patient outcomes [90, 109].

#### Early brain injury: BBB Disruption, neuroinflammation, and cerebral edema

The period spanning the first 72 h after SAH represents a biologically dynamic stage in which the acute mechanical and biochemical insults of aneurysm rupture evolve into a coordinated set of vascular, inflammatory, and metabolic disturbances collectively termed EBI. Central to this stage is the progressive breakdown of the BBB, which arises from endothelial dysfunction, tight junction disassembly, and basal lamina degradation triggered by hemoglobin products, oxidative stress, and inflammatory mediators [102]. Experimental and clinical studies demonstrate early reductions in the expression of claudin-5, occludin, and ZO-1, accompanied by increased activity of matrix metalloproteinases particularly MMP-9 which drives proteolytic cleavage of perivascular extracellular matrix components and further destabilizes endothelial architecture [110]. This loss of barrier integrity permits unregulated flux of plasma proteins, immune cells, and water into the interstitial space, creating the substrate for vasogenic edema and impaired microvascular flow [111].

Neuroinflammation is another defining feature of EBI, emerging within hours of hemorrhage. Blood derived damage associated molecular patterns activate pattern-recognition receptors on microglia, astrocytes, and infiltrating leukocytes, promoting rapid release of pro-inflammatory cytokines such as IL-1β, IL-6, and TNF-α [99]. Transcriptomic and proteomic analyses of cerebrospinal fluid and peri-vascular tissue have consistently highlighted upregulation of inflammatory mediators including S100A8/A9, complement components, and adhesion molecules, underscoring the centrality of myeloid activation and leukocyte trafficking to early inflammatory amplification. These immune responses not only exacerbate BBB leakage but also disrupt neurovascular coupling, impair synaptic function, and potentiate excitotoxicity [101, 112].

Cerebral edema in the EBI window reflects the combined effects of BBB failure, inflammatory cell infiltration, aquaporin dysregulation, and ischemia driven metabolic compromise [102]. Astrocytic swelling, mediated in part by altered AQP4 polarity and oxidative injury, contributes to cytotoxic edema, while vasogenic edema results from protein-rich fluid accumulation in perivascular and interstitial compartments [113]. As edema progresses, intracranial pressure rises, cerebral perfusion pressure declines, and microcirculatory flow becomes increasingly heterogeneous. Mitochondrial dysfunction driven by ROS excess, calcium overload, and impaired oxidative phosphorylation further compromises neuronal and vascular cell energetics, promoting apoptotic and necrotic pathways that intertwine with inflammatory processes to worsen tissue injury [93, 114, 115].

Collectively, BBB disruption, neuroinflammation, and evolving cerebral edema form an interdependent triad that shapes the trajectory of early neurological deterioration after SAH [103]. These mechanisms, now supported by multi omics profiling and real time imaging biomarkers, underscore the need for therapeutics that simultaneously stabilize the vascular endothelium, modulate early immune signaling, and preserve mitochondrial integrity during this critical early period [116].

#### Delayed cerebral ischemia: microcirculatory dysfunction, cortical spreading depolarizations, and immunometabolic remodeling

**Fig. 4:**
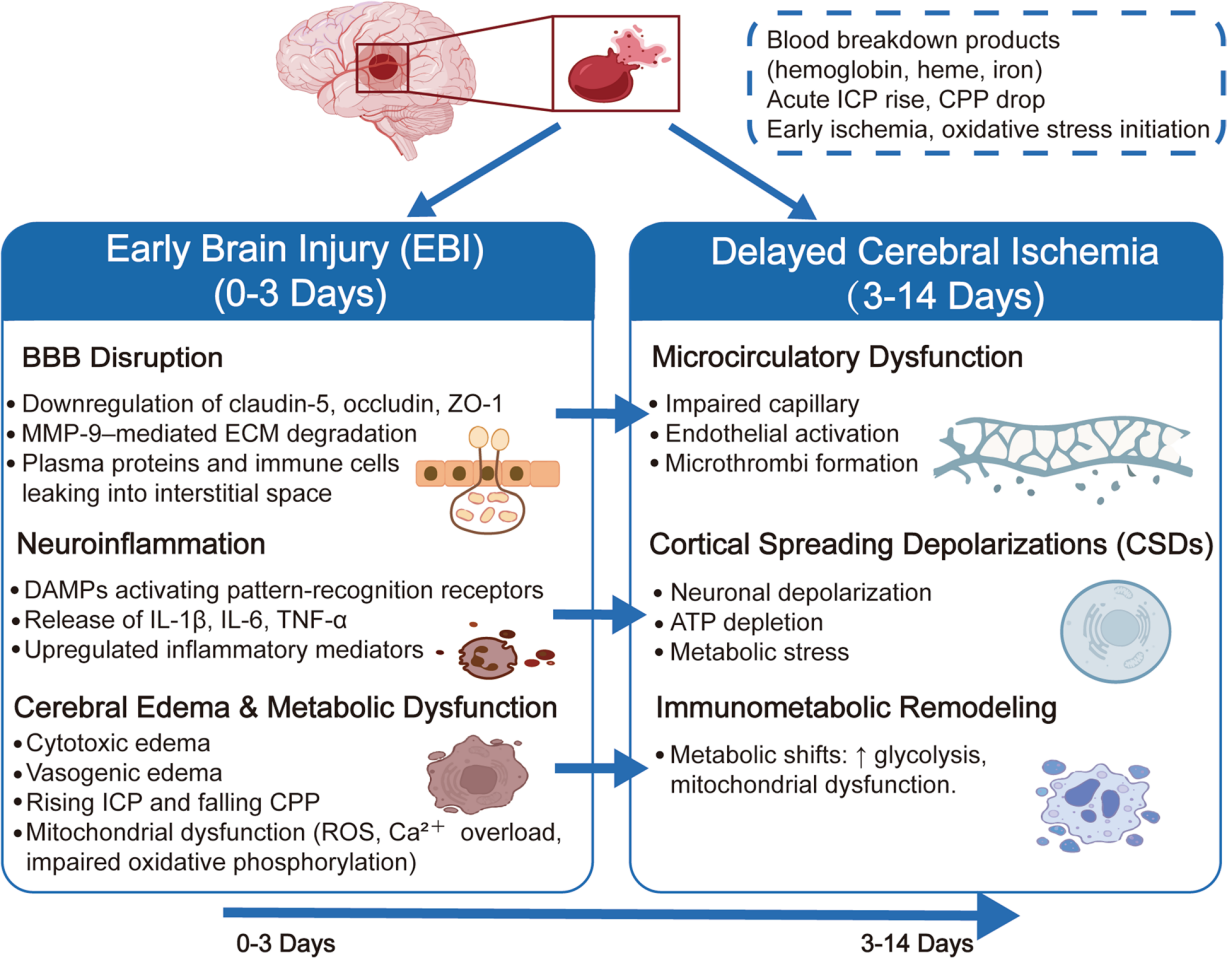
Mechanistic interplay between early brain injury and delayed cerebral ischemia after SAH. This after subarachnoid hemorrhage, EBI and DCI interact with each other. Within the first 72 h after subarachnoid hemorrhage, the disruption of the blood–brain barrier, neuroinflammation, and brain edema are caused by substances from the blood and oxidative stress. As the condition progresses to DCI (3—14 days), microvascular dysfunction, cortical spreading depolarization (CSD), and immune-metabolic changes will exacerbate the ischemic condition. These mechanisms highlight the complexity of DCI and the need for treatment strategies targeting vascular and metabolic pathways to prevent secondary damage

These early pathological events collectively create a vulnerable cerebral vascular and neuroinflammatory environment, which does not recover on its own after the initial injury but instead evolves into a complex chain reaction leading to DCI. To illustrate this temporal continuity and mechanistic correlation, Fig. 4 provides a comprehensive schematic summary. DCI remains an important factor contributing to morbidity and mortality after SAH, typically manifesting between 3 and 14 days after the rupture. Although traditionally DCI is believed to be caused by large vessel CVS, modern evidence emphasizes the crucial role of microcirculation dysfunction, endothelial injury, and neurovascular coupling imbalance, which are often independent of angiographic detected vasospasm [117–119]. Hemoglobin breakdown products, oxidative stress, and inflammatory mediators jointly activate endothelial cells, reduce the bioavailability of NO, and promote microthrombus formation, all of which damage capillary perfusion and regional oxygen supply [120]. High resolution imaging and histopathological studies have consistently shown that these microvascular disorders are closely related to secondary ischemia and the occurrence of focal infarction, even in brain regions supplied by nonspastic arteries [121].

Another factor contributing to DCI is cortical diffuse depolarization (CSD), which is a non-vascular mechanism that exacerbates ischemic damage [122]. CSD is a series of nearly fully depolarized neuronal electrical activity waves that propagate slowly over the cortical surface, causing transient ion imbalances, energy depletion, and metabolic stress in neurons and glial cells [123]. Clinical monitoring studies have shown that recurrent or persistent CSD is strongly correlated with an increased risk of delayed infarction and poor neurological outcomes (especially in areas with concurrent hypoperfusion) [124]. The interaction between CSD and microvascular dysfunction forms a spatial and temporal non-uniform pattern of ischemic damage, which complicates treatment targeting and highlights the complexity of the DCI pathogenesis.

New research evidence further indicates that immune metabolic remodeling plays a significant role in the development of DCI. The infiltration of inflammatory cells, especially that caused by macrophages and neutrophils, promotes local oxidative stress and the production of cytokines [125]. Simultaneous metabolic changes in immune cells and nerve cells, including enhanced glycolysis, mitochondrial dysfunction, and impaired antioxidant defense capacity, exacerbate the brain's susceptibility to ischemia [30, 126]. Proteomic analysis has revealed that during DCI, the expression of chemokines such as MCP-1, CXCL10, and IL-8 in cerebrospinal fluid is significantly upregulated, suggesting that they may serve as early predictive biomarkers and become targets for intervention mechanisms [127–129]. Additionally, matrix metalloproteinases (MMPs), particularly MMP-9, promote the degradation of extracellular matrix, further disrupting the integrity of capillaries and promoting the formation of edema in ischemic areas [130].

In summary, DCI is caused by the interaction of multiple factors such as microcirculation dysfunction, cortical diffuse depolarization, and immune metabolic imbalance [23]. This comprehensive understanding goes beyond the previous single focus on vasospasm, emphasizing the need to develop treatment strategies that can stabilize microvascular flow, regulate cortical excitability, and restore metabolic homeostasis [131]. Such intervention measures may play a crucial role in preventing secondary ischemic injury and improving the long term prognosis of patients with SAH.

## Proteomics and molecular biomarkers in SAH

SAH is accompanied by complex molecular alterations that evolve dynamically and contribute to secondary brain injury [114]. This section focuses on proteomics based investigations of these molecular changes, highlighting the identification and characterization of protein biomarkers associated with key pathological processes, including vascular dysregulation, inflammation, oxidative stress, blood–brain barrier disruption, and neuronal injury [132]. Furthermore, it examines the translational potential of these biomarkers, particularly in relation to risk stratification, early detection of DCI, and the development of targeted therapeutic strategies. A comprehensive understanding of these proteomic signatures is essential for linking underlying mechanisms to clinical outcomes and advancing precision medicine approaches in SAH.

### Key protein biomarker categories

Proteomic and targeted protein studies have clarified that secondary brain injury after SAH is driven by an interacting set of biological processes vascular regulation, inflammation, oxidative stress, BBB disruption, and neuronal injury rather than by isolated molecular events. Large scale profiling efforts have catalogued numerous candidates and, importantly, identified repeatedly observed classes of proteins whose dynamics parallel clinical evolution and complications such as vasospasm and DCI [127, 133]. A concise synthesis of representative markers and their putative roles is provided in Table 1.

**Table 1:** Key protein biomarkers associated with SAH

**Functional Classification**	**Biomarker**	**Sample source**	**Validation Cohort**	**Sample time**	**Trend**	**Ref**
**Inflammation-Related Biomarkers**						
	**IL-6**	CSF	Yes(*n* = 57 SAH, 65 Controls)	The second day of admission	Significantly elevated	[134]
		CSF/ECF/Plasma	Yes(*n* = 38 SAH, 42 controls)	10 days after admission	Significantly elevated(highest IL-6 levels in CSF)	[135]
		Serum	Yes(*n* = 43 SAH, 23 Controls)	10 days after admission	Significantly elevated(most significant at 4th day)	[136]
	**IL-1ra**	CSF	Yes(*n* = 22 SAH, 10 Controls)	1–12 days after admission	Significantly elevated(most significant between days 3 and 10)	[137]
		Serum	Yes(*n* = 58 SAH)	At admission and after 10 days	Sustained elevation	[138]
	**IL-4**	Serum/CSF	Yes(*n* = 210 SAH, 210 Controls)	1–5 days after admission	Significantly elevated	[139]
	**IL-1β**	CSF	Yes(*n* = 81 SAH)	1–3, 4–6, and 7–9 days after admission	Significantly elevated	[140]
		Serum/CSF	Yes(*n* = 15 SAH, 15 Controls)	5 days after admission	Significantly elevated(most significant at 2th day)	[141]
	**S100A8/A9**	Serum	Yes(*n* = 54 SAH, 54 Controls)	48 h after admission	Significantly elevated(most significant at 24–36 h)	[142]
	**TNF-α**	CSF	Yes(*n* = 57 SAH, 65 Controls)	The 2th day of admission	Significantly elevated	[134]
		CSF	Yes(*n* = 81 SAH)	1–3, 4–6, and 7–9 days after admission	Significantly elevated	[140]
	**CRP**	Serum	Yes(*n* = 100 SAH)	10 days after admission	At 0–3 days significantly increases but 4–10 days decreases	[143]
	**CCL5**	CSF/Serum	Yes(*n* = 80 SAH, 41 Controls)	1 and 7 days after admission	Significantly elevated	[144]
	**AIM2**	Serum	Yes(*n* = 127 SAH, 56 Controls)	1, 2, 3, 5, 7 and 10 days after admission	Significantly elevated	[145]
	**OPN**	Plasma	Yes(*n* = 109 SAH)	1–3, 4–6, 7–9, and 10–12 days after onset	Significantly elevated (peaked at days 4–6)	[146]
		CSF/Plasma	Yes(*n* = 44 SAH, 56 Controls)	At admission and the first 24 h from the bleeding and at day 4 and 8	Significantly elevated(peaked at day 8)	[147]
**Oxidative Stress-Related Biomarkers**						
	**TRX**	Serum	Yes(*n* = 132 SAH, 132 Controls)	At admission	Significantly elevated	[148]
	**OLR1**	Plasma	Yes(*n* = 60 SAH, 59 Controls)	6 h of admission	Significantly elevated	[149]
**Vasospasm-Related Biomarkers**						
	**ET**	Plasma	Yes(*n* = 70 SAH, 25 Controls)	28 days after onset	Significantly elevated	[150]
		CSF/Plasma	Yes(*n* = 30 SAH, 10 Controls)	/	Significantly elevated	[151]
	**MMP-9**	Serum	Yes(*n* = 45 SAH, 42 Controls)	1–12 days after admissionor until the onset of vasospasm	Significantly elevated(most significant at 3 days before the onset of CVS)	[152]
		Serum	Yes(*n* = 11 SAH, 20 Controls)	1, 2, 4, 8, 12 days after admission	Significantly elevated	[153]
		CSF/Blood	Yes(*n* = 55 SAH)	14 days after admission	Significantly elevated(most significant at 4–5 days before the onset of blood)	[154]
		Serum	Yes(*n* = 43 SAH, 23 Controls)	10 days after admission	Significantly elevated(most significant at 4th day)	[136]
	**vWF**	Blood	Yes(*n* = 106 SAH)	14 days after admission	Significantly elevated(most significant at 5-9th day)	[155]
		Serum	Yes(*n* = 45 SAH, 42 Controls)	12 days after admission or until the onset of vasospasm	Significantly elevated(most significant at 5th day before the onset of CVS)	[152]
	**VEGF**	Serum	Yes(*n* = 45 SAH, 42 Controls)	12 days after admission or until the onset of vasospasm	Significantly elevated(most significant at 2 days before the onset of CVS)	[152]
	**MPO**	Serum	Yes(*n* = 54 SAH, 5 Controls)	5–25 days after admission (Vasospasm group)	Significantly elevated(most significant at 2 days before the onset of CVS)	[156]
	**HRG**	CSF/Serum	Yes(*n* = 14 SAH, 7 Controls)	0–11 days after onset	Significantly higher (most significant at 48 h)	[157]
	**MCP-1**	CSF/Serum	Yes(*n* = 77 SAH, 14 Controls)	14 days after onset or discharge from the hospital	Significantly elevated	[158]
	**NPY**	CSF	Yes(*n* = 66 SAH)	1–10 days after onset	Significantly higher	[159]
**Blood–Brain Barrier Disruption-Related Biomarkers**						
	**ALP**	Serum	Yes(*n* = 196 SAH, 78 Controls)	1, 3–5, 7–9, 12–14 days after admission	Significantly elevated	[160]
	**Albumin**	Serum	Yes(*n* = 273 SAH)	24 h and 1 week after onset	Significant falling trend (1 week)	[161]
**Neuron Injury-Related Biomarkers**						
	**GFAP**	Serum	Yes(*n* = 67 SAH)	At admission	Significantly elevated	[162]
		Serum	Yes(*n* = 116 SAH)	1, 2, 3, 4, 6, 8 and 10–15 days after admission	Significantly elevated	[163]
		Plasma	Yes(*n* = 105 SAH)	At admission	Significantly elevated	[164]
		Serum	Yes(*n* = 46 SAH, 78 Controls)	24 h after onset and at admission	Significantly elevated	[165]
	**UCH-L1**	CSF	Yes(*n* = 30 SAH)	0–10 days after onset	Significantly elevated	[166]
		CSF	Yes(*n* = 25 SAH)	1, 3, 7, 10 days after onset	Significantly elevated	[167]
		Plasma	Yes(*n* = 105 SAH)	At admission	Significantly elevated	[164]
	**S100B**	Serum	Yes(*n* = 71 SAH, 120 Controls)	24 h, 3 and 7 day after onset	Significantly elevated	[168]
		Serum	Yes(*n* = 67 SAH)	At admission	Significantly elevated	[162]
		Serum	Yes(*n* = 107 SAH)	15 days after admission	Significantly elevated	[169]
		Plasma	Yes(*n* = 48 SAH)	7 days after admission	Significantly elevated(most significant at 5th day)	[170]
		Serum	Yes(*n* = 52 SAH)	1–3 days after admission	Significantly elevated	[171]
		Serum	Yes(*n* = 91 SAH, 25 Controls)	0, 1, 3 days and 10–12 days after onset	Significantly elevated	[172]
	**NSE**	CSF/Serum	Yes(*n* = 20 SAH, 15 Controls)	3, 5, 7 days after onset	Significantly elevated	[173]
		Plasma	Yes(*n* = 105 SAH)	At admission	Significantly elevated	[164]
		Serum	Yes(*n* = 52 SAH)	1–3 days after admission	Significantly elevated	[171]
	**Nesfatin-1**	Plasma	Yes(*n* = 48 SAH, 48 Controls)	At admission	Significantly elevated	[174]
		Serum	Yes(*n* = 62 SAH, 35 Controls)	Unspecified	Significantly elevated	[175]
	**NfL**	CSF	Yes(*n* = 48 SAH)	10–14 days after onset	Significantly elevated	[176]
		Serum	Yes(*n* = 91 SAH, 25 Controls)	0, 1, 3 day and 10–12 day after onset	Significantly elevated	[172]
	**Ngb**	Serum	Yes(*n* = 58 SAH, 27 Controls)	1, 2, 3, 5, 7 days after onset	Significantly elevated(most significant at 2 days)	[177]
	**MBP**	CSF/Serum	Yes(*n* = 28 SAH, 25 Controls)	4–14 days after onset	Significantly elevated(most significant at 4–9 days)	[178]
		Plasma	Yes(*n* = 105 SAH)	At admission	Significantly elevated	[164]
		CSF/Serum	Yes(*n* = 104 SAH)	0–3, 4–6, 9–12 days after onset	Significantly elevated	[179]
	**KLK6**	Serum	Yes(*n* = 13 SAH, 136 Controls)	5–14 days after onset	Significant decrease	[180]
	**Tau-protein**	CMD	Yes(*n* = 22 SAH)	0–8 days after onset	Significantly elevated(most significant at the first 24 h)	[181]
		CDM	Yes(*n* = 26 SAH)	9 days after onset	Significant higher(peak at 72 h after onset)	[182]
		Plasma	Yes(*n* = 105 SAH)	At admission	Significantly elevated	[164]

Inflammatory mediators, including IL-6, TNF-α, IL-1β, IL-8, IL-1ra, IL-4, CRP and S100A8/A9, increase in the early stage after hemorrhage and are associated with vasospasm, DCI risk and poorer prognosis, reflecting the activation of JAK/STAT, MAPK and NF-κB pathways [134, 135, 140, 141, 183–192]. The results of CCL5 are abnormal and require dynamic monitoring [129, 144], while the correlations of AIM2 and OPN in cerebrospinal fluid and plasma vary in specificity and are related to poor prognosis [145–147]. MCP-1 and thrombin markers may indicate neuronal damage, but further validation is needed [158, 193, 194]. Although these markers have information value for monitoring, their diagnostic utility is limited due to sampling variability and limitations of the detection platform, but they are valuable as dynamic indicators of inflammatory load and prognostic models [141, 195–197].

Proteins indicative of oxidative stress and redox imbalance such as thioredoxin (TRX) and oxidized LDL receptor 1 (OLR1) have been associated with disease severity and mortality in single center studies, supporting a mechanistic link between hemoglobin/iron driven ROS production and cellular injury [105, 149, 198–202]. While TRX shows promise when combined with clinical scores, these findings require validation in larger, standardized cohorts before routine application [148, 199, 200].

Endothelial and vasomotor markers, notably ET-1, vWF, VEGF and MMP-9, reflect endothelial activation, matrix remodeling and vasoconstrictive propensity, and have been associated with vasospasm and early ischemic injury [136, 150–156]. Novel candidates (e.g., HRG, NPY, MCP-1, MPO) have emerged from CSF and plasma studies, but heterogeneity of thresholds and small sample sizes constrain immediate clinical translation [157, 159, 203]. Integrative approaches that combine these molecular signals with clinical grading and imaging may improve predictive performance [109, 203, 204].

Markers of BBB disruption and systemic response such as alkaline phosphatase (ALP) and serum albumin capture aspects of endothelial dysfunction and systemic illness burden; albumin reduction on admission has been repeatedly linked to poorer outcomes, whereas ALP findings are inconsistent across populations [160, 161, 205–212]. As such, these markers are most useful within multiparametric panels rather than as solitary predictors.

Neuronal and glial injury markers (GFAP, S100B, NSE, NfL, UCH-L1, MBP, Tau and others) provide relatively specific readouts of parenchymal damage and have shown reproducible associations with functional outcome, with GFAP and S100B being among the most robust and translatable to clinical practice [163, 175–177, 179, 192, 213–215]. Emerging candidates (e.g., Nesfatin-1, Ngb, KLK6) warrant further study but currently offer incremental value primarily within combined biomarker imaging models [172, 174–180, 215–223].

In summary, protein biomarkers supply mechanistic insight and incremental prognostic information but, in isolation, are constrained by interstudy heterogeneity. Their principal translational role at present is as components of multimodal prediction frameworks integrated with clinical scoring, imaging and physiologic monitoring to improve early risk stratification and guide individualized management. Large, multicenter studies with harmonized sampling and assay protocols are required to advance select candidates from promising markers to validated clinical tools.

### Translational potential of proteomic biomarkers

Proteomic studies have enhanced understanding of the molecular events underlying DCI and provide promising translational opportunities. Several protein markers reflect key pathological processes including vasospasm, inflammation, endothelial dysfunction, and neuronal injury that drive secondary brain injury after SAH. A summary of these markers and their potential roles in risk stratification and early intervention is presented in Table 2.

**Table 2:** Protein markers associated with DCI prediction

Proteomic biomarker	Validation Cohort	Sample source	Cut-off Value	Predictor	Sample time	Trend	Ref
**CVS Prediction-Related Biomarkers**							
**ET-1**	Yes(*n* = 70 SAH, 20 Controls)	Plasma	3.1 ± 0.8 pg/ml(0–5 day)	CVS	14 days after admission	Significantly higher	[150]
	Yes(*n* = 23 SAH, 10 Controls)	CSF	Mean 2.45 ± 0.72 pg/ml (at 1 day after surgery)	CVS	10 days after surgery	Significantly higher (most significant at 5 days after surgery)	[224]
	Yes(*n* = 106 SAH)	CSF	7.94 ± 6.47 pg/h(72 h prior DCI development)	CVS	14 days after onset	Significantly higher	[225]
**IL-6**	Yes(*n* = 19 SAH, 12 Controls)	CSF	1207.20 ± 629.14 pg/ml(mean, DID)	CVS	No signify	Significantly higher	[226]
	Yes(*n* = 15 SAH)	CSF	4802 ± 1170 ng/ml	CVS	0–3 days after onset	Significantly higher	[227]
	Yes(*n* = 64 SAH)	CSF	2000 pg/ml(day 4)	CVS	14 days after onset	Significantly higher(most significant at 4 and 5 days after onset)	[228]
	Yes(*n* = 57 SAH, 65 Controls)	CSF	362.56 ± 30.14 pg/ml(mean)	CVS	The second day of admission and during the physical examination	Significantly higher	[134]
	Yes(*n* = 82 SAH)	Serum/CSF	26,291 ± 24,159 pg/ml(mean; DCI)	DCI	4–14 days after onset	Significantly higher (a peak between days 4 and 14 including a maximum on day 5 after SAH.)	[229]
**Clinically Relevant Outcome Prediction-Related Biomarkers**							
**D-dimer**	Yes(*n* = 138 SAH)	Serum	0.445 μg/ml (Fisher grade IV)	CVS, DCI	Admission,1 day after therapy, day 4, day 9, day 14, or discharge from the ICU	Significantly higher(at admission, day 9, and day 14)	[230]
**IL-1ra**	Yes(*n* = 22 SAH, 10 Controls)	CSF	318 pg/ml(mean; poor clinical condition on admission)	Outcome	1–12 days after onset	Significantly higher(peak at 4–10 days after onset)	[137]
	Yes(*n* = 44 SAH)	Serum/CSF	2565.4 ± 802.3 pg/ml(6th day; CFS)	Outcome, Hunt-Hess Grades, GOS	First 2 weeks after onset	Significantly higher(peak at 6th day after onset)	[231]
**TNF-α**	Yes(*n* = 19 SAH, 12 Controls)	CSF	4.38 ± 4.45 pg/ml	Outcome; GOS	At admission	Significantly higher	[226]
	Yes(*n* = 57 SAH, 65 Controls)	CSF	49.68 ± 7.02 pg/ml (mean)	Outcome; Hunt-Hess Grades	The second day of admission and during the physical examination	Significantly higher	[134]
**sTNFR-I**	Yes(*n* = 44 SAH)	Serum/CSF	276.3 ± 142.0 pg/ml(12th day; CSF)	Outcome; Hunt-Hess Grades, GOS	First 2 weeks after onset	Significantly higher	[231]
**Lp-PLA2**	Yes(*n* = 114 SAH)	Serum	200 μg/L(at admission)	Outcome, WFNS grade, mFisher scores	0–2 days after admission	Day0 significantly increased, then decreased	[232]
**NFL**	Yes(*n* = 48 SAH)	CSF	9035 ng/l(median)	Outcome, GOSE	10–14 days after onset	Significantly higher	[176]
**TK**	Yes(*n* = 92 SAH, 92 Controls)	Serum	0.109 mg/mL	Outcome, DCI	At admission	Significantly decreased	[233]
**Ngb**	Yes(*n* = 126 SAH)	Serum	> 8.4 ng/ml(3th day)	Outcome, DCI	Days 1,2,3,5 and 7 after onset	Significantly higher (most significant at 3th day)	[234]

Endothelin-1 (ET-1) and IL-6 exemplify biomarkers associated with CVS, a major contributor to DCI. Elevated ET-1 in plasma or CSF correlates with the onset of vasospasm and delayed ischemic events, particularly within the first week post-hemorrhage [150, 151, 224, 225, 228, 235–237]. Similarly, IL-6 elevations precede radiographic vasospasm and associate with increased DCI risk, although heterogeneity in sampling timing and assay platforms currently limits routine clinical application [134, 226–229, 238].

In addition to vasospasm, inflammatory and neuronal damage markers can also provide more predictive value [239]. D-dimer in cerebrospinal fluid, interleukin-1 receptor antagonist (IL-1ra), and TNF-α reflect the activation of systemic and local inflammation and are related to the severity and functional prognosis of DCI [134, 137, 226, 230, 231]. Metabolic and neuronal markers, including lipoprotein-associated phospholipase A2 (Lp-PLA2) and neurofilament light chain (NfL), further provide risk information. Early elevation indicates subsequent ischemic injury and long-term neurological dysfunction [176, 232, 240]. Emerging molecules such as thymidine kinase (TK) and Ngb respectively indicate endothelial dysfunction and neuroprotective response, which may help enhance individualized prediction of DCI [233, 234].

The translational value of these biomarkers lies in their integration with imaging and physiological monitoring to guide personalized management. Combining molecular signatures with radiographic and hemodynamic data may improve identification of patients at high risk for DCI, optimize timing of therapeutic interventions, and tailor neurocritical care strategies. Future validation in large, multicenter cohorts with standardized sampling protocols will be essential to establish clinical utility and improve patient outcomes after SAH.

## Clinical presentation and diagnosis

The clinical manifestations and routine diagnostic procedures for SAH are mainly based on characteristic symptoms, supplemented by neuroimaging examinations and CSF analysis. Although non-enhanced CT, CT angiography (CTA), digital subtraction angiography (DSA), and lumbar puncture together form a solid framework for confirming the diagnosis and identifying the source of the hemorrhage, each of these examination methods has inherent limitations in terms of sensitivity, time dependence, or invasiveness [241]. In particular, some patients have obvious clinical symptoms but negative or unclear imaging results, highlighting the persistent diagnostic gap in current clinical practice. These challenges indicate the need for supplementary strategies beyond traditional structural imaging examinations. Therefore, recent studies have increasingly focused on molecular biomarkers, using them as a supplement to the conventional diagnostic methods, aiming to improve early detection capabilities, enhance diagnostic accuracy, and provide more pathophysiological insights [242, 243]. Therefore, the following sections will explore the emerging role of biomarkers in the diagnosis of subarachnoid hemorrhage, their integration with imaging methods, and their potential contributions to building a more comprehensive and personalized diagnostic framework.

### Clinical features and standard imaging

**Fig. 5:**
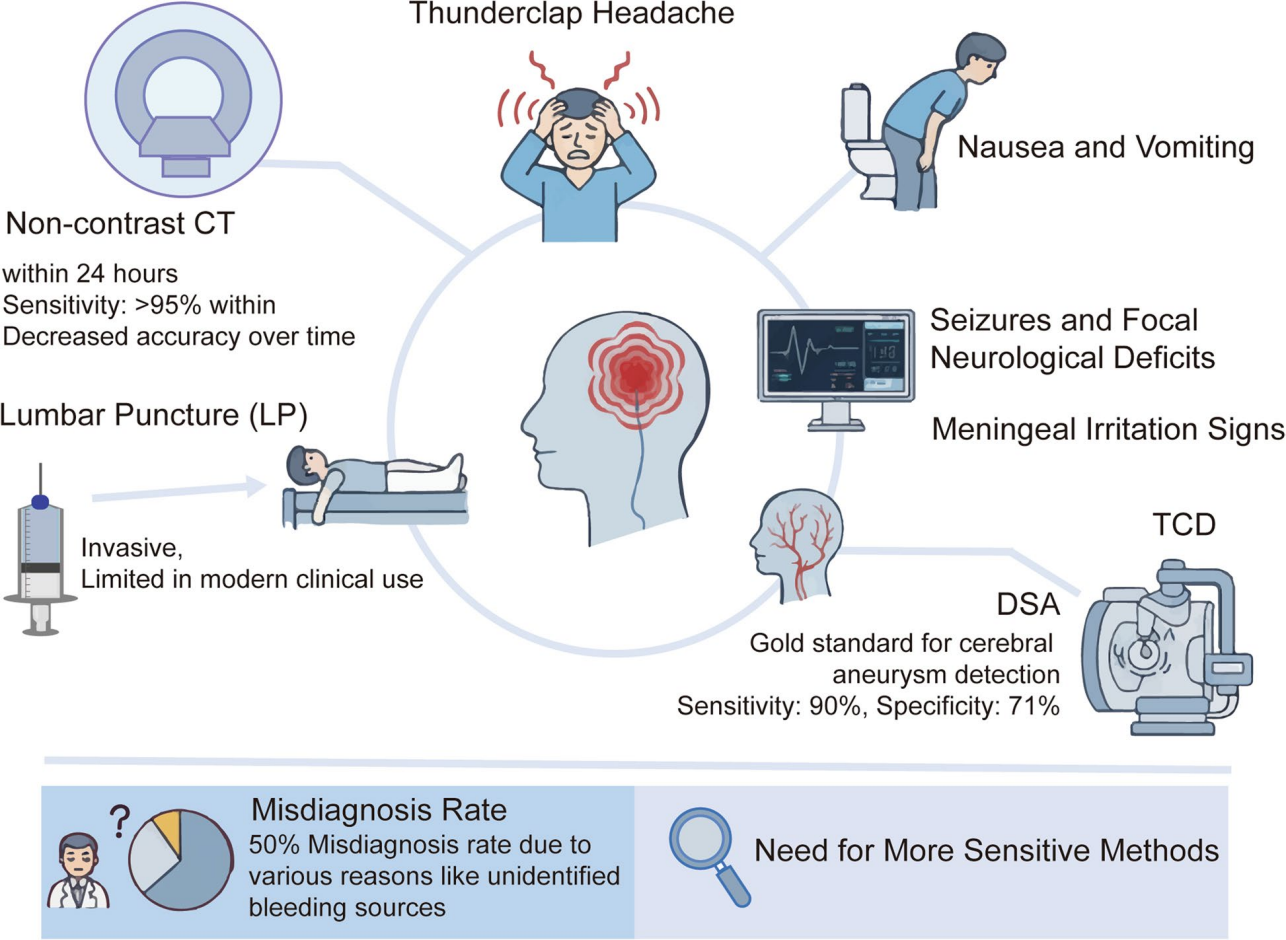
Clinical presentation and diagnostic evaluation pathways for SAH. The key presenting symptoms of SAH such as thunderclap headache, nausea/vomiting, seizures, and meningeal signs and outlines commonly used diagnostic tools, including non-contrast CT, lumbar puncture, DSA, and TCD. Despite the availability of these modalities, SAH continues to have an estimated 50% misdiagnosis rate, underscoring the need for more sensitive and comprehensive diagnostic strategies

SAH typically presents with a sudden, severe “thunderclap” headache, often described as the worst headache in a patient’s life. This hallmark symptom is frequently accompanied by nausea, vomiting, impaired consciousness, and meningeal irritation signs such as neck stiffness [244, 245]. In some cases, seizures or focal neurological deficits may also occur, and their severity is usually correlated with the volume of hemorrhage and secondary complications [246, 247]. An overview of these typical clinical manifestations is illustrated in Fig. 5.

The classical diagnostic workup for SAH integrates neuroimaging and CSF analysis. Non-contrast CT is the first-line imaging modality, with a sensitivity of > 95% in the first 24 h after ictus, though its diagnostic accuracy declines with time [248–254]. When CT findings are inconclusive, lumbar puncture can be performed to detect xanthochromia, although its invasive nature limits routine use in modern clinical practice. CTA is valuable for detecting intracranial aneurysms and is often used as a screening tool or as a complement to DSA, which remains the gold standard despite its invasiveness and risk of procedure related complications [255, 256]. Transcranial Doppler (TCD) ultrasonography is also recommended for monitoring, with reported sensitivity and specificity of 90% and 71% for predicting DCI [257–259]. At present, DSA remains the gold standard for aneurysm detection, though its widespread use is limited by invasiveness and the risk of procedural complications [260]. Nevertheless, about 15% of patients present without an identifiable bleeding source, and the initial misdiagnosis rate may reach 50% [261–263]. These diagnostic limitations underscore the urgent need for more sensitive and earlier adjunctive approaches.

### Molecular biomarkers complementing traditional diagnosis

#### Complementary role in imaging diagnosis

In the diagnosis and monitoring of SAH and its complications, imaging modalities such as CT, CTA, and DSA remain the clinical gold standards. However, these approaches are primarily used to confirm hemorrhage and aneurysm location, while their sensitivity for predicting secondary complications such as EBI or DCI is limited [248–256, 260]. Proteomics, as a molecular level complementary tool, enables dynamic monitoring of protein alterations in CSF or blood and may provide risk stratification earlier than conventional imaging.For example, elevated ET-1 levels have been shown to parallel changes in cerebral blood flow velocity detected by transcranial Doppler (TCD), serving as an early biomarker for vasospasm [264, 265]. Notably, ET-1 rises significantly even before ischemic changes become visible on imaging [266]. In CT-negative patients, proteomics allows detection of low abundance proteins in CSF, capturing subtle pathological alterations, suggesting potential etiologies, and guiding subsequent diagnostic or therapeutic interventions. Similarly, GFAP and S100B proteins associated with neuronal injury and BBB disruption can provide critical diagnostic clues in patients with negative CT findings but high clinical suspicion of SAH, indicating microstructural brain damage or BBB permeability changes and thus supporting further CTA or DSA evaluation. The release kinetics and peripheral detectability of these biomarkers have been systematically validated in studies of neurotrauma and BBB dysfunction [267] Moreover, CRP elevation has been consistently linked with CVS, DCI, and poor outcomes. A systematic review and meta analysis confirmed a significant association between early CRP elevation and DCI, while multicenter and single center studies suggested that CRP peaks within 3–4 days after onset have stronger predictive value for unfavorable neurological outcomes compared to a single admission measurement, supporting its use as a complementary monitoring marker alongside imaging and clinical scales [195, 268].

#### The application of biomarkers in early diagnosis

Recent studies suggest that proteomic biomarkers may serve as adjunctive diagnostic tools to complement imaging modalities. GFAP and S100B, which reflect astrocytic and neuronal injury, can detect subtle brain damage and BBB disruption, particularly in CT-negative patients [269–271]. In addition, CRP, while a nonspecific inflammatory marker, has been correlated with the severity of cerebral edema and vasospasm observed on CT/MRI, indicating potential value in risk stratification [272]. Incorporating these molecular biomarkers into the diagnostic workflow may enhance early recognition of pathological processes and provide a biochemical complement to traditional radiological methods.

### Multimodal integration and personalized diagnosis

Proteomic studies have demonstrated that several key proteins, including VEGF, MMP-9, and multiple inflammatory mediators, undergo time dependent dynamic changes after SAH. These molecules contribute to increased vascular permeability, BBB disruption, and basement membrane degradation, while also showing potential as predictive biomarkers for complications such as CVS and DCI [273]. For instance, VEGF upregulation has been strongly associated with enhanced vascular permeability and cerebral edema formation. Both animal and translational studies support the causal link between VEGF inhibition and reduced brain edema/permeability [274]. Cross modal validation has already been achieved in other neurological and oncological conditions: MRI-based radiomics and radio genomics models can predict VEGF expression and angiogenic activity in tumors, providing a methodological framework for “imaging protein” mapping [275]. In cervical cancer, radiomic signatures have been shown to predict VEGF expression levels [276], in gliomas, dynamic contrast enhanced MRI (DCE-MRI) has been used to infer the expression of angiogenesis-related proteins [277].

In cases of SAH, diffuse high signals are often observed on T2 weighted magnetic resonance imaging (MRI), which reflect brain edema and potential ischemic conditions. Recent clinical studies and reviews have shown that the early increase in BBB permeability is associated with T2 high signals and diffusion abnormalities, which are related to poor prognosis. This indicates that they have value as imaging surrogate indicators in risk stratification of complications [102, 278, 279]. Based on these insights, combining imaging omics with proteomics may enable earlier and non-invasive assessment of key protein dynamics, thereby detecting the risks of edema, vasospasm, and DCI before imaging, providing a basis for personalized therapeutic interventions [280, 281].

## Clinical management and therapeutic strategies

The clinical treatment of subarachnoid hemorrhage has evolved into a structured and comprehensive strategy that includes acute physiological stabilization, clear aneurysm repair, and adjunctive drug intervention, aiming to prevent secondary brain injury [282]. These established methods have significantly improved early survival rates and reduced the risk of severe rebleeding; however, they remain largely supportive and symptomatic. It is important to note that the occurrence of delayed complications such as cerebral vasospasm and delayed cerebral ischemia is still leading to adverse consequences, reflecting the incomplete control of the underlying pathophysiological cascade. This gap between clinical intervention and the complexity of the disease highlights the necessity of more mechanism-driven treatment strategies [283, 284]. In this context, increasing efforts are being made to identify key molecules and signaling pathways related to SAH progression, which may become potential targets for future intervention. Therefore, the following section will explore novel experimental therapies targeting specific pathways and the latest progress based on proteomics research, which provide new insights into the molecular basis of subarachnoid hemorrhage and its therapeutic applications.

### Standard clinical and surgical treatment

**Fig. 6:**
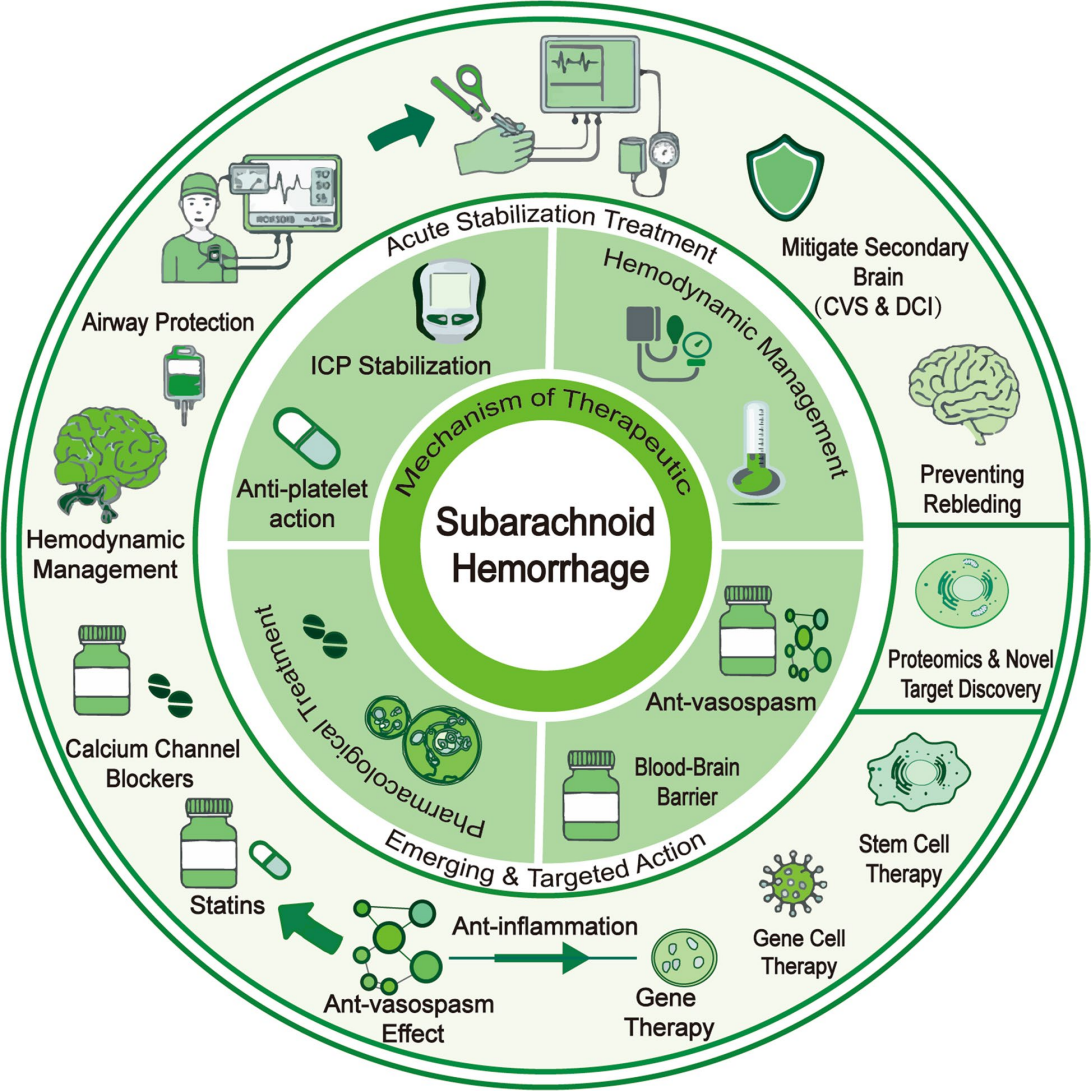
Key points in the management of SAH. For patients with SAH, a comprehensive and continuous approach is required for effective clinical management. Before the onset of SAH, efforts should be made to control risk factors, such as hypertension. After SAH occurs, early recognition of symptoms and prompt emergency transportation are crucial. Upon hospital admission, timely and efficient collaboration among relevant departments is essential, with careful selection of treatment strategies. These interventions play a significant role in improving the patient's prognosis

The standard management of SAH encompasses a combination of acute stabilization, aneurysm repair, and pharmacological prevention of secondary complications (see Fig. 6). Initial stabilization in the intensive care unit focuses on airway protection, intracranial pressure (ICP) control, hemodynamic management, and prevention of rebleeding [282, 285, 286]. Aneurysm treatment is achieved through either microsurgical clipping or endovascular coiling, both of which are well established strategies to secure the ruptured vessel and reduce the risk of recurrent hemorrhage [287]. Alongside these cornerstone interventions, pharmacological therapy plays a crucial supportive role, with the primary goal of reducing secondary brain injury, particularly CVS and DCI.

Current pharmacological management focuses on preventing secondary brain injury, with most agents targeting CVS and DCI. Currently available drugs mainly include calcium channel blockers, antiplatelet agents, anti-inflammatory drugs, and statins. Although these treatments can improve outcomes to some extent, their overall efficacy remains limited, and no agents directly targeting the primary pathophysiological mechanisms have been established. Calcium channel blockers represent the only class of drugs currently recommended by international guidelines for DCI prevention. Among them, nimodipine has been consistently shown in randomized controlled trials to significantly reduce the incidence of neurological deficits [288]. Its mechanism of action extends beyond selective cerebral vasodilation, potentially including stabilization of the BBB, attenuation of cortical spreading depolarizations (SDs), and modulation of inflammation and injury related proteins such as S100B and GFAP [289–291]. Other agents, including nicardipine and magnesium sulfate, have shown potential for CVS alleviation but lack large scale clinical validation [289]. Antiplatelet therapy plays a role in preventing microthrombus formation and improving microcirculatory perfusion, thereby lowering the risk of DCI [292–294]. Cilostazol, a phosphodiesterase III inhibitor with antiplatelet, antiproliferative, and vasodilatory effects, has demonstrated benefits in reducing DCI incidence and improving clinical outcomes [295, 296]. Its effects may involve suppression of adhesion molecules such as ICAM-1 and vWF. Dual antiplatelet therapy (e.g., aspirin plus clopidogrel) also shows potential, though the issue of clopidogrel resistance highlights the need for platelet function testing or genetic profiling to enable individualized therapy [294].

Anti-inflammatory treatment remains another crucial therapeutic approach. Corticosteroid drugs such as dexamethasone can downregulate proinflammatory cytokines including IL-6 and TNF-α, thereby alleviating secondary brain damage [297]. However, the combined therapy of dinoprostone and fasudil has been reported to regulate the NF-κB and VCAM-1 signaling pathways, improve endothelial function, and alleviate cognitive impairment [298]. Proteomic analysis further indicates that inflammatory proteins including IL-1ra, sTNFR-I, MMP-9, ICAM-1, and VCAM-1 may serve as biomarkers for monitoring the response to anti-inflammatory drugs and identifying new targets [299]. At the same time, statin drugs with anti-inflammatory and anti-vasospasm effects have also been studied. Some studies have shown that statin drugs can reduce the incidence of CVS by downregulating CRP, IL-1β, and S100A8/A9, and improve neurological function [300]. However, the evidence from large-scale randomized controlled trials is still inconsistent, and their routine use has not yet been supported.

Overall, the current drug treatments still have limited efficacy. Their main role is to provide support and preventive treatment rather than targeting the fundamental pathological process of SAH. Proteomics offers a new perspective by elucidating molecular pathways and changes in dynamic biomarkers, which helps identify new therapeutic targets and provides molecular basis for evaluating the efficacy of existing drugs.

### Pathway targeted experimental therapies and research advances

Although some progress has been made in the treatment of SAH, significant clinical improvement is still limited by factors such as individual patient differences, unclear definition of the treatment window, and the lack of validated targets for mechanisms. Proteomics research is beginning to fill this gap by identifying changes in pathway levels related to inflammation, oxidative stress, vascular dysfunction, blood–brain barrier integrity, and neuronal damage, thereby providing more precise guidance for translational drug development. The study emphasizes that inflammatory-related proteins, oxidative stress regulators, vascular mediators, and neuronal injury markers are key factors and potential therapeutic targets for the progression of SAH [150, 152, 154, 301]. Table 3 summarizes the representative proteins associated with SAH complications and their targeted mechanisms.

**Table 3:** Potential drug targets and progress in experimental research

Target	Drug Name	Mechanism of Action	Study Type	Drug Dose (if applicable)	Research or Clinical Status	Remarks	Ref
**Inflammatory targets**							
sgp130	None	Blocks classical IL-6 signaling	Clinical Trial	No data available	Preclinical research	No drug development; tested in the lab	[302]
IL-6 pathway	p-STAT3 inhibitor	Reduced NFΚB, TLR4, and nitro tyrosine	Animal Study	No data available	Animal model studies	Potential research on targeting inflammatory following SAH	[188]
IL-1	IL-1ra	Decreased concentration of CSF IL-6	Clinical Trial	500 mg bolus, then a 10 mg/kg/hour infusion for 24 h	Phase II clinical trials	Potential research on targeting inflammation following SAH	[303]
IL-1	IL-1ra	Significantly reduced levels of IL-6 and C-reactive protein	Clinical Trial	100 mg,twice daily, subcutaneously	Phase III clinical trials	Subcutaneous IL-1Ra is safe and well tolerated in SAH. It is effective in reducing peripheral inflammation	[304]
S00A8/A9	None	Anti-S100A8/A9 antibodies or its antagonists	Clinical Trial	No data available	Preclinical research	No drug development; tested in the lab	[142]
TNF-α	SB203580	Inhibiting the phosphorylation of p38 MAPK in the basilar artery and by suppressing the increase in TNF-α	Animal Study	6.7 μmol/kg, each day, injected into the cisterna magna	Animal model studies	Studied only in SAH animal models, capable of inhibiting post-SAH vasospasm	[305]
CCR1	Met-RANTES	Selective CCR1 antagonist	Animal Study	20 ng, 50 ng, or 150 ng, intranasal administration	Animal model studies	Studied only in SAH animal models, capable of inhibiting post-SAH neuroinflammation	[306]
NF-κB	Curcumin	Restricting NF-κB activation	Animal Study	150 mg/kg, injection	Animal model studies	Studied only in SAH animal models, capable of inhibiting post-SAH inflammatory response	[307]
NLRP3	MCC950	Reduced NLRP3 inflammasome expression	Animal Study	10 mg/kg, injected intraperitoneally	Animal model studies	MCC950 alleviated SAH-induced EBI by suppressing	[308]
NLRP3	MCC950	Reduced NLRP3 inflammasome expression	Animal Study	40 mg/kg, injected intraperitoneally	Animal model studies	MCC950 and other NLRP3 inhibitors could be promising tools in the development of therapeutics for EBI and DCI	[309]
NLRP3	SalB	Blocking NLRP3 inflammasome and promoting SIRT1 signaling	Animal Study	20 or 40 mg/kg, injected	Animal model studies	Studied only in SAH animal models, capable of inhibiting post-SAH neuroinflammation	[310]
**Oxidative stress targets**							
mitochondrial apoptotic pathway	SS31	Significantly reduced MDA levels and restored the activities of GPx and SOD in the temporal cortex	Animal Study	2/5/10 mg/kg, injected intraperitoneally	Animal model studies	Potentially beneficial in post-SAH Oxidative Stress research	[311]
Keap1/Nrf2/PHB2 pathway	Mitoquinone (MitoQ)	Inhibited oxidative stress-related neuronal death by activating mitophagy via Keap1/Nrf2/PHB2 pathway	Animal Study	1/3/9 mg/kg injected intraperitoneally	Animal model studies	Potentially beneficial in post-SAH Oxidative Stress research	[312]
Nfr2	RTA408	Increases in Nrf2 and decreases in NF-κB and iNOS	Animal Study	0.5/1/1.5 mg/kg/day, injected intraperitoneally	Animal model studies	Potentially beneficial in post-SAH Oxidative Stress research	[313]
**Vasospasm targets**							
ET(A/B)receptors	TAK-044	Blocking the binding of ET-1 to ETA and ETB receptors	Clinical Trial	50 mg given three times per day, injection	Phase II clinical trials	Reduces the frequency and severity of cerebral vasospasm following SAH	[314]
ETRA	Clazosentan(AXV-034343)	Blocking the binding of ET-1 to ETA receptors	Clinical Trial	0.2/0.4 mg/kg/hour, injection	Phase IIa clinical trials	Reduces the frequency and severity of cerebral vasospasm following SAH	[315]
ETRA	Clazosentan−2	Blocking the binding of ET-1 to ETA receptors	Clinical Trial	5 mg/hour, injection,	Phase III clinical trials	Clazosentan at 5 mg/h had no significant effect on mortality and vasospasm-related morbidity or functional outcome	[316]
MMP-9	SB-3CT	Inhibit the activity of MMP-9	Animal Study	0.3 mg per animal, injection into the cisterna magna	Animal model studies	Potential research on targeting vasospasm following SAH	[317]
vWF	rADAMTS‐13	Regulate the activity of vWF	Animal Study	3460 U/kg, injection	Animal model studies	Reduce early brain injury after experimental SAH	[318]
VEGF	BVE	By binding to VEGF, it blocks its interaction with VEGF receptors	Animal Study	5 mg/kg,,injected intraperitoneally	Animal model studies	Potential research on targeting vasospasm following SAH	[319]
**Blood–brain barrier target**							
Albumin	ALISAH II	Reducing BBB damage and minimizing secondary brain injury	Clinical Trial	1.25 g/kg/day ×7 days, injection	Phase III clinical trials	Potentially beneficial in post-SAH BBB repair research	[320]
	None	Reducing BBB damage and minimizing secondary brain injury	Animal Study	0.02–1.0 g/kg, injection	Animal model studies	Potentially beneficial in post-SAH BBB repair research	[321]
**Neuronal injury marker**							
Nesfatin-1	None	Inhibition of neutrophil infiltration and the subsequent release of inflammatory mediators	Animal Study	10 μg/kg, injection	Animal model studies	Potential neuroprotective effects following SAH	[322]
	None	Inhibition of neutrophil infiltration and the subsequent release of inflammatory mediators	Animal Study	5, 10, 20 μg/kg, injection	Animal model studies	Potential neuroprotective effects following SAH	[323]
GFAP	Cottonseed oil (CSO)	Reducing microglial and astrocytic activation and inflammation	Animal Study	1.3 ml/kg, subcutaneous injection,	Animal model studies	Potential to alleviate neuroinflammatory damage following stroke	[324]

Inflammation is an important mechanism leading to secondary brain injury and delayed cerebral ischemia. Proteomics data consistently indicate that IL-6, IL-1 family cytokines, TNF-α, S100A8/A9, and components of the STAT3/TLR4/NF-κB signaling pathway are highly effective therapeutic nodes [325]. Elevated IL-6 levels are one of the most common findings in SAH. Experimental studies have shown that knocking out IL-6 or blocking the IL-6/STAT3 signaling pathway through drugs can reduce vasospasm and improve neurological function [188]. Soluble gp130 (sgp130) further regulates IL-6 bypass signal transduction and provides an additional intervention point [302]. Selective inhibition of STAT3 can alleviate the activation of NF-κB, TLR4, and nitrotyrosine, thereby reducing inflammatory related neuronal damage [188]. The IL-1 pathway has shown early translational potential. IL-1 receptor antagonists (IL-1Ra) can cross the BBB and show good safety characteristics in SAH trials. The research by Singh and Galea shows that it can significantly reduce IL-6 levels in cerebrospinal fluid and plasma [303, 304, 326]. IL-4 also shows neuroprotective effects in the preclinical stage [139].

The amplification of S100A8/A9 after SAH enhances TLR4 signaling and is associated with the clinical severity, providing a potential biomarker-target combination for the development of future antiagents [142]. The endothelial dysfunction induced by TNF-α also aggravates CVS and DCI; inhibiting p38 MAPK with SB203580 can suppress the upregulation of TNF-α and improve the results of vasospasm [305]. CCR1 blockade (Met-RANTES) interferes with JAK2/STAT3 signaling and alleviates neuroinflammation [306]. Natural compounds such as curcumin can also inhibit multiple targets of NF-κB and show beneficial effects in reducing vasospasm after SAH [307].

Mechanistically, the NLRP3 inflammasome has gained attention as an upstream initiator of EBI. The selective inhibitor MCC950 reduces inflammasome activation and downstream cytokine release, with consistent efficacy demonstrated by Luo et al. and subsequently confirmed by Dodd et al. [308, 309]. Salvianolic acid B further suppresses NLRP3 activity through SIRT1 enhancement, offering an additional anti-inflammatory axis [310]. Collectively, inflammation targeted strategies already span the preclinical clinical continuum and remain among the most mature translational directions in SAH.

Oxidative stress is a major determinant of both EBI and DCI, resulting from hemoglobin degradation, mitochondrial dysfunction, and excessive ROS generation [327]. Mitochondrial injury, in particular, represents one of the earliest triggers of EBI and has become a high priority therapeutic target [328, 329]. Mitochondria-targeted antioxidants such as SS-31 attenuate neuronal death by stabilizing mitochondrial membranes and suppressing caspase-dependent apoptosis [311]. MitoQ exerts neuroprotection through activation of the Keap1/Nrf2/PHB2 axis, enhancement of mitophagy, and reduction of ROS-induced neuronal loss, with benefits extending into long-term neurological recovery [312]. The Nrf2 activator RTA408 reduces oxidative stress and inflammation, alleviates vasospasm, and improves functional scores in SAH models [313]. These findings reinforce oxidative-stress modulation particularly Nrf2-centered pathways as a promising, mechanism aligned therapeutic strategy. Integration of proteomic screening and patient stratification may accelerate clinical translation.

CVS (Cerebral Perfusion Insufficiency) remains the main cause of DCI (Cerebral Ischemia Reperfusion Injury) and poor prognosis. The treatment approaches for this condition mainly focus on endothelin signaling, extracellular matrix regulation, and microthrombosis mechanisms. Endothelin (ET) plays a central role in sustained vasoconstriction. Blocking ET (A/B) receptors using TAK-044 can reduce the incidence of DCI and has good tolerance [314, 315]. Chlorazosentan showed a reduction in vasospasm in angiography in the Phase IIa study [316]. However, the III phase trial did not show any improvement in mortality or functional prognosis, and the systematic review again emphasized that although there was an improvement in imaging, there was a lack of clear clinical benefits [330, 331]. These differences highlight the need for more precise patient screening and the necessity of combined pathway targeting strategies. The MMP-9 that is rapidly upregulated after SAH participates in vascular remodeling and CVS (Cerebral Perfusion Insufficiency). Using SB-3CT to inhibit its activity can significantly reduce vasospasm in experimental models [317]. Abnormal vWF promotes microthrombosis; recombinant ADAMTS-13 can alleviate early ischemia and improve microcirculation flow [318]. Anti-VEGF strategies such as bevacizumab can reduce pathological angiogenesis and vascular remodeling in SAH models [319]. Although the prospects are promising, most are still in the early translational stage, so strict clinical validation is needed.

BBB disruption is a defining pathological event in EBI. Albumin therapy has shown early feasibility: a phase I study demonstrated good tolerability in SAH patients [320]. while animal models indicate reductions in cerebral edema, neuronal death, and improvement in neurological scores [321]. BBB stabilization therefore represents an actionable yet underdeveloped therapeutic direction warranting further clinical investigation.

Nesfatin-1, a BBB permeable peptide, has demonstrated consistent neuroprotective properties across SAH models, including reduction of apoptosis, oxidative stress, and inflammatory mediator release, along with attenuation of BBB disruption [322, 323, 332, 333]. Its dual function as a biomarker and therapeutic agent aligns with precision medicine frameworks’ related pathways may also offer neuroprotective potential; evidence from ischemic models suggests that modulation of astrocytic activation (e.g., via cottonseed oil) reduces neuroinflammation and glial overactivation [324]. though this requires validation in SAH settings.

## Outcomes and prognosis

### Traditional predictors

The clinical outcomes of SAH are influenced by multiple factors, among which the traditional grading system remains the most widely used risk stratification tool. The Hunt-Hess grading system based on the patient's neurological condition at admission and the WFNS grading system are closely related to mortality and functional prognosis, reflecting the severity of the disease and having relatively high clinical reliability [334]. The Fisher grading system assesses CVS and DCI by evaluating the amount of hemorrhage and the distribution of blood clots on CT, and is an important imaging based predictor [335]. Moreover, a large amount of clinical evidence indicates that the amount of hemorrhage, the occurrence of rebleeding, and the development of CVS/DCI are the main determinants of long term prognosis after SAH [4, 336, 337]. Although these traditional indicators are widely used in clinical practice, they largely rely on imaging and clinical manifestations, thus presenting issues of subjectivity and limited sensitivity. This highlights the need for supplementary molecular biomarkers to provide a more objective and reliable prognosis assessment.

### Molecular biomarkers as prognostic tools

In recent years, proteomics has provided a novel molecular perspective for the prognosis prediction of SAH. Multiple proteins have been confirmed to be closely related to poor prognosis. Glial fibrillary acidic protein (GFAP), as a typical marker of astrocyte injury, significantly increases in the serum after SAH, and large-scale cohort studies and individual studies have confirmed that its increase is consistent with the adverse neurological outcome 6 months later. Another neuroglial protein, S100B, has been proven to be related to rebleeding, DCI, and mortality. Meta-analysis further confirmed its reliability as a prognostic indicator [338–340]. Ubiquitin C-terminal hydrolase-L1 (UCH-L1), a neuron-specific protein, increases in both cerebrospinal fluid and plasma after SAH; the increase in plasma UCH-L1 is independently associated with adverse neurological outcomes, and when UCH-L1 levels are included in the multi-indicator detection panel, its level is associated with vasospasm and infarction risk, thereby enhancing the prediction of long-term functional prognosis [341]. TRX is a key antioxidant protein, and its serum level is positively correlated with the severity score at admission (WFNS, modified Fisher score) in patients with SAH, and has been identified as an independent predictor of 6-month mortality, highlighting the role of redox imbalance in long term prognosis [148]. Neuron specific enolase (NfL) is a sensitive marker of axonal injury, and its levels in cerebrospinal fluid and serum continue to increase after SAH, and have a strong association with long term functional outcomes. Research evidence has developed from early cerebrospinal fluid studies to recent serum-based analyses, confirming its prognostic value; however, its independent predictive role needs to be verified in larger scale, multi center cohort studies [342, 343].

Overall, GFAP and S100B exhibit the most consistent findings across studies and hold substantial translational potential, while UCH-L1, TRX, and NfL provide additional information for constructing multimarker, multimodal prognostic models. Future work should prioritize standardized sampling protocols, cross-platform validation, and integration of proteomic markers with clinical grading systems and imaging data into prospective prognostic frameworks [204, 239].

## Conclusions and future directions

SAH remains a life-threatening cerebrovascular emergency in which rapid aneurysmal rupture triggers a cascade of molecular and microvascular disturbances that current therapies only partially mitigate. Although advances in aneurysm treatment and neurocritical care have improved early survival, secondary injury driven by inflammation, endothelial dysfunction, BBB breakdown, and impaired microcirculation continues to determine long-term neurological outcomes. Recent progress in proteomics has deepened our understanding of these mechanisms. Key biomarkers such as IL-6, GFAP, S100A8/A9, ET-1, and TNF-α reflect distinct phases of EBI and DCI. Meanwhile, activation of IL-6/STAT3, MMP/NF-κB, NLRP3 inflammasome, and ET-1/ETR pathways provides a mechanistic basis for clinical deterioration, including cerebral edema, metabolic crisis, and microvascular ischemia. These insights offer a molecular framework for refining risk stratification and identifying therapeutic windows. However, clinical translation remains challenged by limited reproducibility, heterogeneous sampling, and the absence of validated biomarker thresholds. Future work must prioritize multicenter, standardized proteomic studies and integrate molecular markers with imaging, physiological monitoring, and clinical grading scales. AI driven multimodal models may further enable precise prediction of complications and individualized treatment strategies. With continued interdisciplinary collaboration, proteomics-guided approaches have the potential to reshape SAH care supporting earlier diagnosis, optimized intervention timing, and improved long term neurological outcomes.

## Data Availability

Not applicable.

## Abbreviations

SAH: Subarachnoid Hemorrhage
DCI: Delayed Cerebral Ischemia
EBI: Early Brain Injury
CVS: Cerebral Vasospasm
LC–MS: Iquid chromatography mass spectrometry
GBD: Global Burden of Disease GBD
DALYs: Disability adjusted life years
ASIR: Age standardized incidence rate
UI: Uncertainty interval
LMICs: Low- and middle-income countries
SDI: Sociodemographic index
WFNS: World Federation of Neurosurgical Societies
ROS: Reactive oxygen species
GOS: Glasgow Outcome Scale
mRS: Modified Rankin Scale
GCS: Glasgow Coma Scale
DAMP: Damage-associated molecular pattern
CSI: Cortical spreading ischemia
CSF: Cerebrospinal Fluid
ECF: Extracellular Fluid
BBB: Blood–Brain Barrier
CNS: Central Nervous System
NND: Neurological Deficits
LC–MS: Liquid Chromatography-Mass Spectrometry
2D-GE: Two-Dimensional Gel Electrophoresis
LC–MS/MS: Liquid Chromatography-Tandem Mass Spectrometry
iTRAQ: Isobaric Tags for Relative and Absolute Quantification
DIA-MS: Data-Independent Acquisition Mass Spectrometry
SWATH-MS: Sequential window acquisition of all theoretical fragment ion spectra mass spectrometry
DDA: Data-dependent acquisition
SPR: Surface Plasmon Resonance
AI: Artificial intelligence
MS/MS: Tandem Mass Spectrometry
IEF-LC: Isoelectric Focusing-Liquid Chromatography
NSCLC: Non-Small Cell Lung Cancer
ICP: Intracranial Pressure
CRP: C-Reactive Protein
ET-1: Endothelin-1
NfL: Neurofilament light chain
S100A8/A9: Calprotectin
MMP-9: Matrix Metalloproteinase-9
OLR1: Oxidized low-density lipoprotein receptor 1
oxLDL: Oxidized low-density lipoprotein
DIND: Delayed ischemic neurological deficits
VEGF: Vascular Endothelial Growth Factor
vWF: Von Willebrand Factor
ET: Endothelin
IL-6: Interleukin-6
IL-8: Interleukin- 8
IL-1: Interleukin- 1
IL-1ra: Interleukin-1 receptor antagonists
IL-4: Interleukin-4
IL-1β: Interleukin-1β
TNF-α: Tumor Necrosis Factor-alpha
AA: Arachidonic Acid
CCL5: C–C chemokine Ligand 5
OPN: Osteopontin
AIM2: Absent in Melanoma 2
TF: Tissue Factor
TAT: Thrombin-Antithrombin III complex
TRX: Thioredoxin
MPO: Myeloperoxidase
HRG: Histidine-Rich Glycoprotein
MCP-1: Monocyte Chemoattractant Protein-1
NPY: Neuropeptide Y
ALP: Alkaline phosphatase
GFAP: Glial Fibrillary Acidic Protein
NSE: Neuron-Specific Enolase
MBP: Myelin Basic Protein
Ngb: Neuroglobin
UCH-L1: Ubiquitin C-terminal Hydrolase L1
KLK6: Kallikrein-related Peptidase 6
CSI: Corticosteroides Inhalados
MMPs: Matrix Metalloproteinases
DID: Delayed Ischemic Deficits
sTNFR-I: Soluble TNF Receptor type I
Lp-PLA2: Lipoprotein-associated Phospholipase A2
NFL: Neurofilament
TK: Tissue kallikrein
SD: Spreading Depolarization
ICAM: Intercellular Adhesion Molecule
VCAM: Vascular Cell Adhesion Molecule
TLR4: Toll-Like Receptor 4
CCR1: C–C Chemokine Receptor 1
NLRP3: Pyrin domain-containing Protein 3
SalB: Salvia miltiorrhiza B
ROS: Reactive Oxygen Species
MitoQ: Mitoquinone
iNOS: Inducible Nitric Oxide Synthase
ETRAs: ET Receptor Antagonists
DINDs: Delayed Ischemic Neurological Deficits
rADAMTS-13: Recombinant ADAMTS-13
BEV: Bevacizumab
CSO: Cottonseed oil
GM: Gray Matter
WM: White Matter
WMI: White Matter Injury
DSA: Digital Subtraction Angiography
CTA: Computed Tomography Angiography
CT: Computed tomography
TCD: Transcranial Doppler
CBFV: Cerebral Blood Flow Velocity
